# Spatiotemporal Variations in Antarctic Protistan Communities Highlight Phytoplankton Diversity and Seasonal Dominance by a Novel Cryptophyte Lineage

**DOI:** 10.1128/mBio.02973-21

**Published:** 2021-12-14

**Authors:** Maria Hamilton, Martina Mascioni, Elisabeth Hehenberger, Charles Bachy, Charmaine Yung, Maria Vernet, Alexandra Z. Worden

**Affiliations:** a Ocean EcoSystems Biology Unit, GEOMAR Helmholtz Centre for Ocean Research Kiel, Kiel, Germany; b Ocean Sciences Department, University of California Santa Cruz, Santa Cruz, California, USA; c Monterey Bay Aquarium Research Institute, Moss Landing, California, USA; d División Ficología, Facultad de Ciencias Naturales y Museo, Universidad Nacional de La Plata, La Plata, Argentina; e Consejo Nacional de Investigaciones Científicas y Técnicas (CONICET), Ciudad Autónoma de Buenos Aires, Argentina; f Integrative Oceanography Division, Scripps Institution of Oceanography, University of California San Diego, La Jolla, California, USA; g Max Planck Institute for Evolutionary Biology, Plön, Germany; University of California, Irvine

**Keywords:** Antarctic fjords, phytoplankton diversity, community structure, protists, Southern Ocean

## Abstract

The Andvord fjord in the West Antarctic Peninsula (WAP) is known for its productivity and abundant megafauna. Nevertheless, seasonal patterns of the molecular diversity and abundance of protistan community members underpinning WAP productivity remain poorly resolved. We performed spring and fall expeditions pursuing protistan diversity, abundance of photosynthetic taxa, and the connection to changing conditions. 18S rRNA amplicon sequence variant (ASV) profiles revealed diverse predatory protists spanning multiple eukaryotic supergroups, alongside enigmatic heterotrophs like the Picozoa. Among photosynthetic protists, cryptophyte contributions were notable. Analysis of plastid-derived 16S rRNA ASVs supported 18S ASV results, including a dichotomy between cryptophytes and diatom contributions previously reported in other Antarctic regions. We demonstrate that stramenopile and cryptophyte community structures have distinct attributes. Photosynthetic stramenopiles exhibit high diversity, with the polar diatom Fragilariopsis cylindrus, unidentified Chaetoceros species, and others being prominent. Conversely, ASV analyses followed by environmental full-length rRNA gene sequencing, electron microscopy, and flow cytometry revealed that a novel alga dominates the cryptophytes. Phylogenetic analyses established that TPG clade VII, as named here, is evolutionarily distinct from cultivated cryptophyte lineages. Additionally, cryptophyte cell abundance correlated with increased water temperature. Analyses of global data sets showed that clade VII dominates cryptophyte ASVs at Southern Ocean sites and appears to be endemic, whereas in the Arctic and elsewhere, Teleaulax amphioxeia and Plagioselmis prolonga dominate, although both were undetected in Antarctic waters. Collectively, our studies provide baseline data against which future change can be assessed, identify different diversification patterns between stramenopiles and cryptophytes, and highlight an evolutionarily distinct cryptophyte clade that thrives under conditions enhanced by warming.

## INTRODUCTION

The West Antarctic Peninsula (WAP) is considered to be among the most climate-sensitive regions on Earth (1). The ocean here has high levels of primary productivity that support a variety of benthic and pelagic life, including many marine mammals, particularly in some of the glacial-marine fjords that pepper this complex region (2–4). Unlike lower-latitude oceans, where both cyanobacteria and photosynthetic unicellular eukaryotes contribute to primary production via photosynthesis, the WAP food web is underpinned solely by photosynthetic protists. However, the taxa that dominate these communities and the environmental factors that shape them are not well resolved. Only a few studies have examined the molecular diversity and phylogenetic relationships across phytoplankton groups or the heterotrophic protists that consume them in the context of seasonal (5) or spatial variability (6) of this ecosystem. Most research on WAP primary producers has been performed using microscopy and pigment-based analyses (7). These studies of the open-water period (rather than the ice-covered period) indicate that austral summer phytoplankton blooms are typically dominated by diatoms or cryptophytes and that blooms of small flagellates and cryptophytes follow the initial diatom bloom stage (2). The haptophyte alga Phaeocystis and other small flagellates are also present (8, 9), although the species comprising the latter have been difficult to identify (10). In nearshore environments, blooms by the small flagellated prasinophyte Pyramimonas sp. and by unidentified unarmored dinoflagellates have been also reported (11, 12).

A striking longer-term trend has been reported for WAP phytoplankton communities, in which there appears to be a shift toward cryptophyte dominance overall (13). Based on multiyear pigment analyses on transects across the region, this shift is thought to be linked to increased warming (13). Phytoplankton communities have been addressed using the 18S rRNA gene to examine the entire microbial eukaryote community at a general level (6, 14–18). Most recently, WAP transect data indicated that a single amplicon sequence variant (ASV) dominated the cryptophyte community at the majority of stations, spanning multiple years, with occasional offsets in peak relative abundances with the known Antarctic cryptophyte Geminigera cryophila (19). Overall, several primarily nonmolecular studies have suggested that general phytoplankton community dynamics in the WAP are shaped by the timing of sea ice retreat and extent of surface water stratification (8, 9, 13). However, there is a paucity of contextualized repeat sampling of molecular diversity and other parameters that currently hinders understanding of phytoplankton dynamics in this region.

Phytoplankton community dynamics are perhaps even less well resolved within the glacial-marine fjords that are common to the WAP coastline. These narrow inlets have steeply elevated sides and at least one tidewater glacier at their terminus (20). Complexity contributed by the presence of multiple fjords is overlain by seasonal changes and localized differences in the input of melted sea ice. Variations in fjord productivity levels also exist, as well as differences in how the interface between the cryosphere and ocean manifests. Moreover, WAP fjords are known to be colder than Arctic fjords, resulting in generally weaker meltwater influences (21). This is important in shaping ecology in the WAP and its fjords because lower meltwater inputs result in reduced upper ocean stratification and reduced inner fjord turbidity, conditions that generally enhance phytoplankton growth and the productivity of polar fjords. One WAP fjord that has been noted for its high primary productivity and an abundance of diverse megafauna is Andvord Bay (22, 23). Five phytoplankton groups have been reported in Andvord Bay and the coastal WAP based on high-performance liquid chromatography (HPLC) pigment analyses; specifically, cryptophytes, diatoms, prasinophytes, dinoflagellates, and in particular “unidentified small phytoflagellates” have been called out as important in Andvord Bay and in other WAP regions (9, 12, 13, 21, 23). Unfortunately, the molecular diversity and taxonomic composition of these small phytoplankton species is currently unknown, although it is important for connecting trends to acclimatization and evolutionary processes relevant to future change.

Here, we examine the molecular diversity of unicellular eukaryotes in Andvord Bay to enhance our understanding of the protistan community responsible for its productivity and the resulting aggregations of feeding mammals (23). Our studies were performed in two seasons within the open-water period, austral spring and fall, and included sampling within and beyond the fjord. High-throughput amplicon (V9 18S rRNA) sequencing (24) was used to characterize diversity of both photosynthetic and heterotrophic protists. We then further characterized the photosynthetic community using V1-V2 16S rRNA primers that recover both bacterial and plastid-derived sequences and are more reflective of photosynthetic organismal relative abundances than 18S rRNA gene amplicons due to more constrained 16S gene copy numbers (25, 26). This was combined with flow cytometric cell enumeration, which provided a quantitative understanding of phytoplankton community gradients in the fjord, in addition to diversity assessments and their association with seasonal changes. After recognizing a seemingly novel cryptophyte amplicon sequence variant (ASV), we performed full-length gene sequencing and field microcopy studies to establish its phylogenetic relationships and morphology. These studies revealed an uncultured cryptophyte that forms a distinct clade separate from that containing the canonical Antarctic cryptophyte Geminigera cryophila (27). Not only is the novel lineage present in multiple Antarctic settings, but its abundance is correlated with water temperature, a factor that is directly connected to climate change.

## RESULTS

### Molecular diversity of microbial eukaryotes in Andvord Bay and beyond.

**FIG 1:**
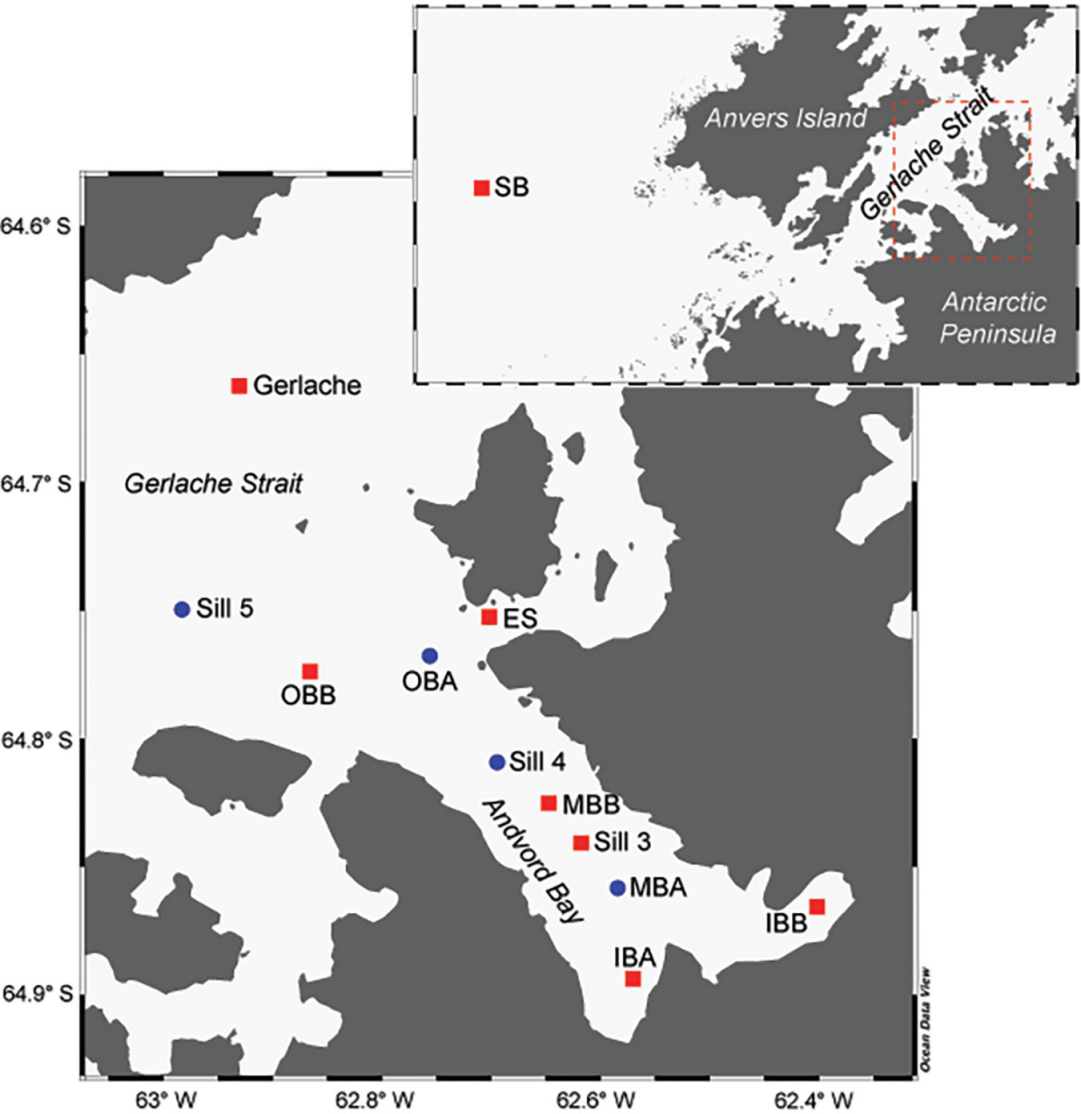
Sampling stations in the present study, including Station B (SB) on the Antarctic continental shelf and stations within and adjacent to Andvord Bay in the Gerlache Strait. The map in the foreground shows a broader view of the west coast of the Antarctic Peninsula and Anvers Island, with the red box outlining the map of Andvord Bay shown in the background. Red squares indicate stations that were sampled and analyzed during both the austral spring and fall, while blue circles represent stations that were only sampled and analyzed during the austral spring.

We analyzed the molecular diversity of the microbial eukaryotic community in Andvord Bay and the adjacent Gerlache Strait. The Gerlache Strait is the conduit to Bransfield Strait and the continental margin surrounding the South Shetland Islands (28) (Fig. 1). V9 18S rRNA sequencing of four austral spring (November to December 2015) and two austral fall (April 2016) surface samples resulted in 1,256,577 total amplicons after quality control (see Table S1 in the supplemental material). Rarefaction analysis indicated that the depth of sequencing was saturated for all samples (Fig. S1A). Average species richness was higher in austral spring (396 ± 83) than in fall (226 ± 41) samples, although differences in sample numbers could influence these results. Shannon diversity estimates were similar between austral spring (3.49 ± 0.28) and fall (3.27 ± 0.34). Of the 885 total V9 18S ASVs, 627 were unique to spring samples, and 58 were only detected in fall samples. Some of the ASVs “unique” to spring reached up to 4% of total amplicons within a single sample, while many other seasonally unique ASVs were low in relative abundance (<0.6% of total amplicons), including all those present in fall samples.

10.1128/mBio.02973-21.1 FIG S1 Rarefaction curves of the 18S rRNA gene amplicon sequence variants (ASVs) (A) and the V1-V2 16S rRNA plastid ASVs (B). Austral spring samples are indicated with a blue dot, and fall samples with a red dot. Sequence numbers are provided in Table S1. Download FIG S1, TIF file, 0.3 MB. Copyright © 2021 Hamilton et al. 2021 Hamilton et al. https://creativecommons.org/licenses/by/4.0/ This content is distributed under the terms of the Creative Commons Attribution 4.0 International license.

10.1128/mBio.02973-21.8 TABLE S1 Station names and depths where samples were taken for DNA filtration, the numbers of total V9 18S rRNA gene amplicon sequences, total V1-V2 16S rRNA gene amplicon sequences, and plastid sequences. Download Table S1, XLSX file, 0.04 MB. Copyright © 2021 Hamilton et al. 2021 Hamilton et al. https://creativecommons.org/licenses/by/4.0/ This content is distributed under the terms of the Creative Commons Attribution 4.0 International license.

**FIG 2:**
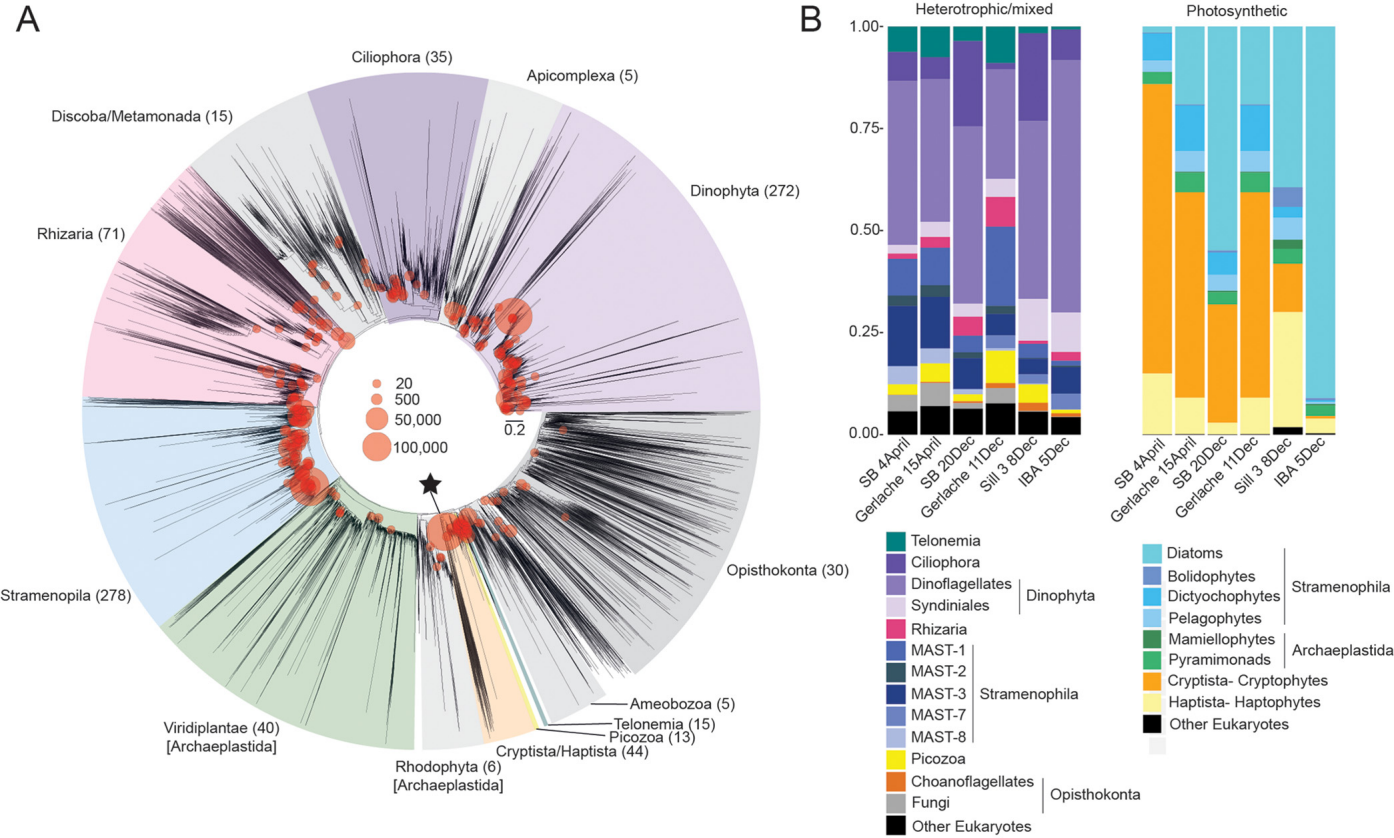
Eukaryotic diversity and distributions at Andvord Bay and Gerlache Strait stations. (A) Depiction of reference maximum-likelihood phylogenetic construction (based on near-full-length 18S rRNA gene sequences [see Materials and Methods] onto which 18S V9 amplicon sequence variants [ASVs] from the austral spring and fall samples were mapped using published methods [29]). The size of the red circle is proportional to the number of amplicons (within the ASV assigned to the specific node) out of the total number of quality-controlled amplicons generated. Major eukaryotic groups are highlighted, and the number of ASVs represented is given in parentheses next to each group label. The black star indicates the node representing cryptophytes. (B) Relative 18S rRNA gene amplicon frequency for this subset of samples, based on Qiime 2 classification, with a subsequent partitioning based on a literature review of nutritional types. The “heterotrophic/mixed category” includes strict heterotrophs, sequences classified into broad taxonomic groups that include both heterotrophic and mixotrophic (capable of phagotrophic nutrition and photosynthesis) representatives, and groups where the nutritional mode could not be identified (i.e., dinoflagellates). Only groups represented at ≥10% relative contributions in one or more samples are shown. Note that MAST-12 was also present but placed in the “other eukaryotes” classification in panel b, along with other ASVs in taxonomic groups of low relative abundance (<10% in all samples) and ASVs classified as “unidentified” based on Qiime 2 (see Table S2 in the supplemental material). The “other eukaryotes” category under photosynthetic eukaryotes comprised ASVs in taxonomic groups that were either present at low relative abundance (<10% in all samples) or lacked a taxonomic classification beyond “Archaeplastida.” All samples represented were also analyzed using the V1-V2 16S rRNA gene region (see Fig. 4).

To gain an overview of the protists present in our samples, we used published methods (29) that involved mapping the 18S ASVs to a curated reference maximum-likelihood phylogenetic reconstruction that employed nearly full-length 18S rRNA gene sequences. The reference reconstruction contained representative sequences from all major eukaryotic groups from both cultured taxa and environmental studies. Our Antarctic 18S ASVs mapped to 242 nodes and demonstrated that each of the major eukaryotic groups in the phylogenetic reconstruction was present in the fjord (Fig. 2A). The most diverse groups were the Dinophyta and Stramenopila, comprising 272 and 278 ASVs, respectively, and spanning 55 and 44 nodes (Fig. 2A). The Cryptista and Haptista (30), represented here solely by cryptophytes and haptophytes, contained the most abundant single ASV. Furthermore, all cryptophyte ASVs were placed at a single node, suggesting high ASV identity (Fig. 2A).

10.1128/mBio.02973-21.9 TABLE S2 List of all 18S and 16S amplicon sequence variants (ASVs) and their taxonomic/nutritional categorization based on Qiime 2 (18S) or PhyloAssigner (16S) classification. Sequences from ASVs mentioned within the text are also included. Download Table S2, XLSX file, 0.04 MB. Copyright © 2021 Hamilton et al. 2021 Hamilton et al. https://creativecommons.org/licenses/by/4.0/ This content is distributed under the terms of the Creative Commons Attribution 4.0 International license.

We next delineated protistan consumer ASVs from those of primary producers (24) by using a compilation of literature-based information. Eight eukaryotic groups were classified as photosynthetic and 13 as frequently heterotrophic or of mixed nutritional modes (Fig. 2B). Lineages with members that are considered either heterotrophic or mixotrophic, defined here as the capability for photosynthesis and phagotrophy, were included in the heterotrophic/mixed category. Because the V9 did not allow confident delineation of photosynthetic, mixotrophic, and heterotrophic dinoflagellates, all were placed in the heterotrophic/mixed category. Multiple lineages of the Telonemia, Stramenopila, Alveolata, and Rhizaria (TSAR) supergroup assemblage also belong to this category, with high relative abundances of rhizarians, ciliates, dinoflagellates, syndiniales, and marine stramenopiles (MASTs). The latter comprised MAST-1, MAST-2, MAST-3, MAST-7, and MAST-8, which were important contributors to the heterotrophic/mixed community during both austral spring and fall, based on relative amplicon abundance. Finally, picozoans, choanoflagellates, and some fungal taxa were also detected and were generally present during both seasons.

Several different eukaryotic lineages were represented among the clearly photosynthetic portion of the community. The major groups were Viridiplantae (green algae within the supergroup Archaeplastida) and lineages within the Stramenopila, Cryptista, and Haptista. Among green algae were 16 mamiellophyte ASVs and 5 pyramimonad ASVs. Within the Stramenopila, 151 different diatom ASVs, 9 bolidophytes, 12 dictyochophytes, and 9 pelagophytes collectively contributed to overall relative abundances (Fig. 2B). Cryptophytes comprised up to 71% of total photosynthetic 18S rRNA gene amplicons. In contrast to the many diatom ASVs, cryptophytes were represented by 22 18S ASVs (Fig. 2B), 20 of which had contributions totaling <0.8% of total cryptophyte 18S ASVs (Table S2). A highly dominant ASV was present, _18S_ASV1, representing 93% of all cryptophytes in the samples, while the second most relatively abundant taxon (_18S_ASV32) contributed just 4%.

### Cryptophyte abundance.

**FIG 3:**
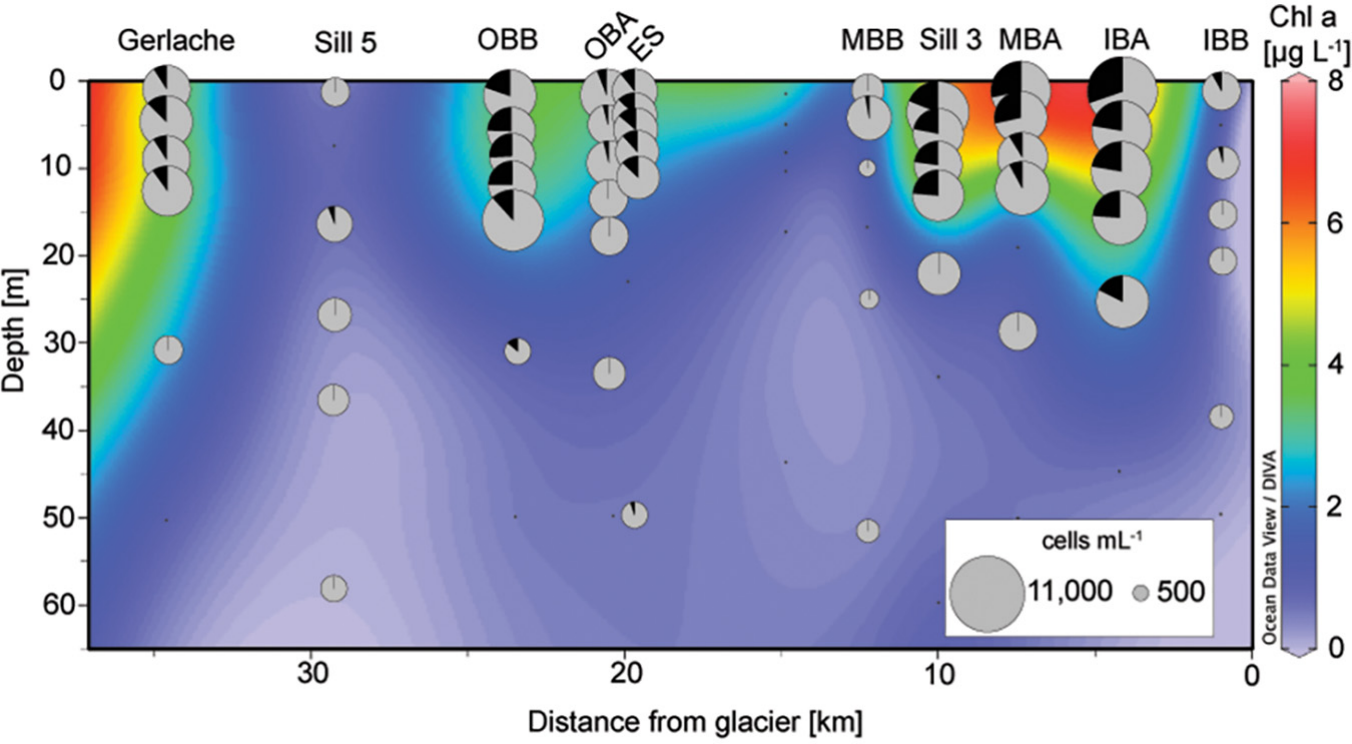
Flow cytometry-based phytoplankton abundances across austral spring depth profiles. Cross section of the upper 65 m of the spring transect showing chlorophyll *a* (Chl *a*) concentration in the background with cell abundance overlaid (pie charts). Black represents the proportion of total cells that were identified as cryptophytes, while gray represents all other photosynthetic cells. The size of each pie chart is scaled to the total number of cells enumerated.

Our next aim was to determine the actual abundances of cryptophytes, which can be unambiguously identified based on their relative size and the phycoerythrin (PE) pigment they contain (31). Cyanobacterial populations, including Synechococcus, were not identified. Quantification of cryptophyte abundance was important because we observed contrasting patterns of cryptophyte and diatom ASVs, with respective relative dominance by one or the other. These patterns could arise as a function of how relative abundances are generated. For example, if one taxon increases in relative abundance, the other might have either decreased or stayed the same in terms of cellular abundance but will appear to decline in amplicon analyses. Using flow cytometry, we observed the highest total phytoplankton abundances in austral spring, corresponding with chlorophyll *a* (Chl *a*) results (Fig. 3 and Table S3), while fall cell abundances and Chl *a* concentrations were low, with maximum measurements of ∼1,303 cells · mL^−1^ (flow cytometry) and 0.80 μg Chl *a* · L^−1^. The highest concentrations in spring were observed in the upper 20 m at each station, reaching a maximum of 10,204 cells · mL^−1^ at Inner Basin A (IBA) (2 December 2015, 1.5 m; Fig. 3). Variability in cell abundance occurred throughout the fjord and adjacent waters, with a mean of 4,465 cells · mL^−1^ (±2,294, *n* = 42) within the fjord, and 5,511 cells · mL^−1^ (±1,482, *n* = 26) outside the fjord.

10.1128/mBio.02973-21.10 TABLE S3 Flow cytometry-based counts of total eukaryotic phytoplankton cells and those that contain phycoerythrin (attributed here to cryptophytes; see Olson et al. [31]) from spring and fall stations. Estimates of biomass (Pg carbon · mL^−1^ seawater) are included as well. Download Table S3, XLSX file, 0.04 MB. Copyright © 2021 Hamilton et al. 2021 Hamilton et al. https://creativecommons.org/licenses/by/4.0/ This content is distributed under the terms of the Creative Commons Attribution 4.0 International license.

Cryptophytes were detected by cytometry at all spring stations except Station B (SB) and had the highest abundances within the fjord. These values corresponded well with microscopy-based confirmation of cryptophyte observations (*r* = 0.92, *P* = 3.2 × 10^−16^), although absolute counts were systematically lower for the latter (Fig. S2). The cryptophytes observed with microscopy all corresponded to a singular morphotype. The greatest number of cryptophytes was found at IBA (2 December 2015, 1.5 m), which featured the highest number of total phytoplankton cells, >30% of which were cryptophytes (i.e., >3,000 cryptophyte cells · mL^−1^; Fig. 3) and ranked second among stations in terms of Chl *a* concentrations. In the upper 60 m, the abundance of cryptophytes and other photosynthetic cells enumerated by flow cytometry were positively correlated with each other (Fig. S3A). The total number of photosynthetic cells was positively correlated with glacial meltwater at this depth range, but the cryptophytes did not show a significant linear relationship with meltwater (Fig. S3B and C). Cryptophyte abundance was positively correlated with temperature and Chl *a* in the upper 60 m (Fig. S3D and E).

10.1128/mBio.02973-21.2 FIG S2 Relationship between cryptophyte cells enumerated with flow cytometry versus those enumerated with light microscopy. Download FIG S2, TIF file, 0.2 MB. Copyright © 2021 Hamilton et al. 2021 Hamilton et al. https://creativecommons.org/licenses/by/4.0/ This content is distributed under the terms of the Creative Commons Attribution 4.0 International license.

10.1128/mBio.02973-21.3 FIG S3 The relationships between photosynthetic cells enumerated with flow cytometry in the upper 60 m of the water column, including cryptophyte cell abundance versus abundance of noncryptophyte cells (A), total cell abundance versus glacial meltwater fraction (B), cryptophyte abundance versus glacial meltwater fraction (C), cryptophyte abundance versus water temperature (D), and cryptophyte abundance versus chlorophyll *a* (Chl *a*) (E). Download FIG S3, TIF file, 0.5 MB. Copyright © 2021 Hamilton et al. 2021 Hamilton et al. https://creativecommons.org/licenses/by/4.0/ This content is distributed under the terms of the Creative Commons Attribution 4.0 International license.

### Plastid diversity across the fjord.

**FIG 4:**
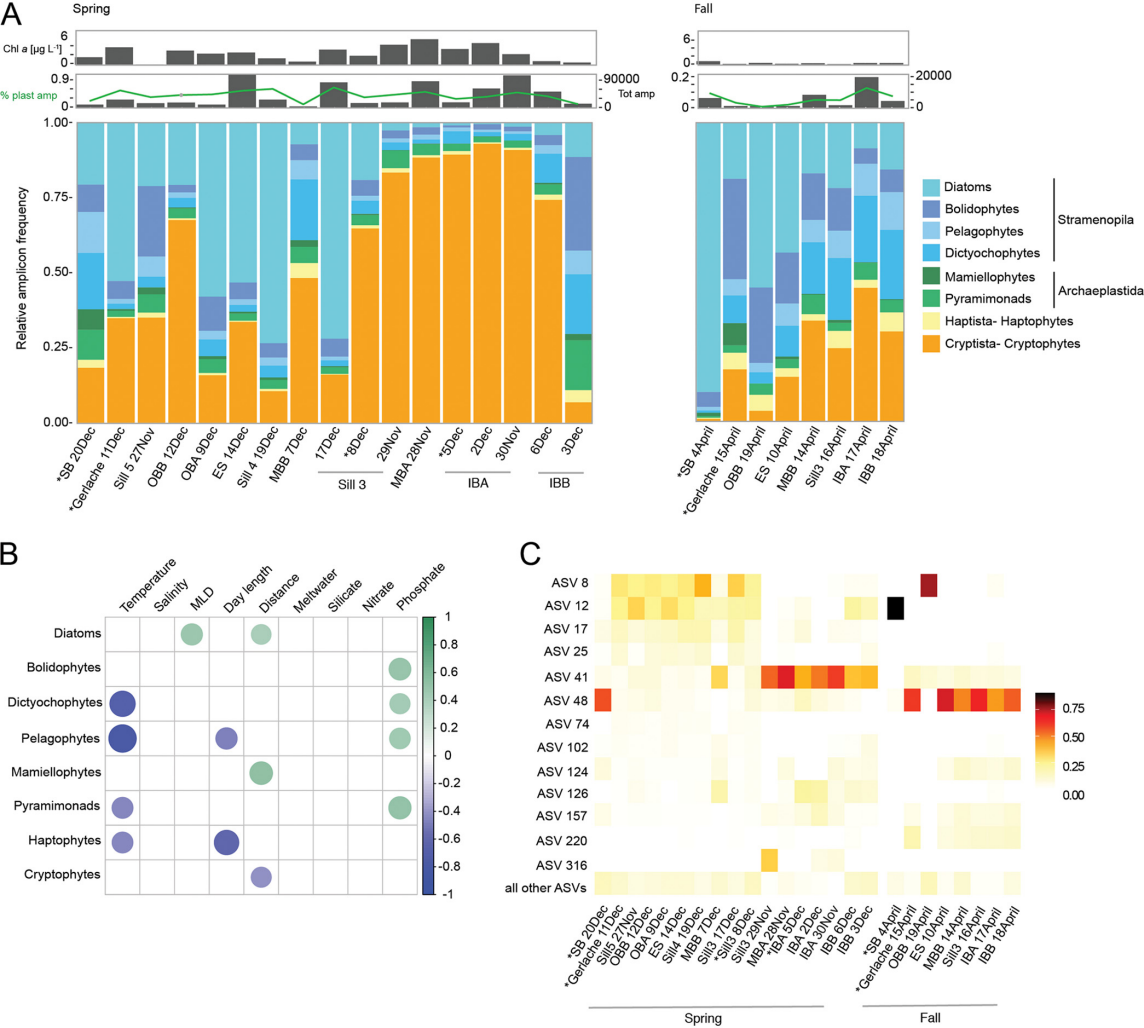
Phytoplankton diversity, distribution, and environmental relationships. (A) Relative 16S rRNA gene amplicon frequency for samples taken during austral spring and fall, based on PhyloAssigner classification. The topmost gray bar plot represents Chl *a* concentrations corresponding to each DNA sample. The lower gray bar plot shows the total number of plastid amplicons sequenced with the percentage of total amplicons that were identified as plastid sequences overlaid as a light green line. An asterisk (*) indicates samples that were also analyzed by 18S rRNA amplicon sequencing (see Fig. 2). (B) Heatmap showing the significant Pearson’s correlations (*P* ≤ 0.05) between environmental factors and relative amplicon frequency of phytoplankton taxa. Pairwise correlations without an assigned colored dot represent correlations that are not significant. (C) Heatmap of the distribution of diatom ASVs as the percentage of total PhyloAssigner-classified diatom amplicons. Shown are the top 10 ASVs, as well as ASVs that formed >10% of the total diatom amplicons in at least one sample. The less frequently observed diatom ASVs were summed and are represented here as “all other ASVs.”

The contrast between the diversity found within most phytoplankton groups and the lack therein of relatively abundant cryptophytes led us to further investigate these WAP primary producers. Using more spatially resolved sampling to search for potential gradients in community structure, we next examined phytoplankton diversity based on plastid-derived 16S rRNA gene ASVs. Rarefaction analyses showed that 16S ASV saturation was reached in the 25 surface samples sequenced (Fig. S1B). The numbers of plastid sequences recovered ranged from 5,043 to 157,703 in spring samples and 1,025 to 19,698 in fall (Table S1), out of 182,191 ± 65,051 and 91,466 ± 59,257 average amplicon sequences, respectively. The number of plastid-derived sequences and relative percentages out of all 16S amplicons (including bacteria) was correlated with Chl *a* concentrations (*r* = 0.71, *P* = 6.3 × 10^−5^; Fig. 4A). Average Shannon diversity examined at the plastid ASV level was not significantly different between spring (2.39 ± 0.54) and fall (2.42 ± 0.54). Furthermore, while no relationship was detected between phytoplankton diversity and meltwater percentages, phytoplankton diversity did exhibit a weak but positive response to distance from the glacial terminus (*r* = 0.46, *P* = 0.03).

To examine phylogenetic relationships between phytoplankton community members and identify specific taxa, sequences were characterized via statistical placement on reference 16S rRNA phylogenetic reconstructions (32) previously developed for analysis of cyanobacteria and eukaryotic phytoplankton (26). The results showed representatives from the same eukaryotic “supergroups” (30) as 18S rRNA ASV analyses, specifically, Viridiplantae (Archaeplastida), Stramenopila, Cryptista, and Haptista (Fig. 4A). As expected based on prior studies (33), cyanobacterial sequences were not detected, although they would be recovered using the primers employed if present (see, e.g., references 34 and 35). The Viridiplantae were represented by two main lineages, pyramimonads and mamiellophytes. Four eukaryotic phytoplankton groups were identified within the Stramenopila, specifically, diatoms, pelagophytes, dictyochophytes, and bolidophytes. Finally, the Cryptista and Haptista were represented only by cryptophytes and haptophytes, respectively. Among the general trends observed was, again, contrasting dominance by diatoms or cryptophytes, particularly in the austral spring (Fig. 4A).

### Phytoplankton-environmental connections and intrataxon diversity.

Among the Viridiplantae, pyramimonad ASVs had higher relative amplicon abundances, except on 15 April 2016 at Gerlache (Fig. 4A). Their relative amplicon abundances were negatively correlated with temperature and positively correlated with increased phosphate concentrations (Fig. 4B). The four pyramimonad ASVs were assigned to a single last common ancestor of Prasinophyte class I node; more precise classification was not possible because 16S rRNA gene sequences from characterized taxa are not available. However, 9 Mamiellophyceae ASVs were detected, three belonging to the picoeukaryote Micromonas polaris and 6 to Bathycoccus. In both seasons, mamiellophytes contributed <10% of total plastid amplicons, reaching maximum contributions of 6.8% in the austral spring and 7.4% in fall, and similarly low numbers of Micromonas cells were detected by quantitative PCR (qPCR) (Fig. S4). Spring and fall Mamiellophyceae relative abundances were positively correlated with distance from the glacier (*r* = 0.53, *P* = 6.0 × 10^−3^) (Fig. 4B).

10.1128/mBio.02973-21.4 FIG S4 Minimum *Micromonas* cells · mL^−1^, estimated using quantitative PCR (qPCR) for a subset of austral spring samples. Error bars show the standard deviation of three replicates. Download FIG S4, TIF file, 0.3 MB. Copyright © 2021 Hamilton et al. 2021 Hamilton et al. https://creativecommons.org/licenses/by/4.0/ This content is distributed under the terms of the Creative Commons Attribution 4.0 International license.

Diatoms were the most highly represented stramenopiles (Fig. 4A) and also showed the greatest diversity of the phytoplankton groups detected, with a total of 66 16S ASVs. Several diatom ASVs had high nucleotide identity to known species, and some were detected only at specific stations, while others presented a more consistent background presence (Fig. 4C). _16S_ASV8 was one of the most relatively abundant diatoms in austral spring in the outer regions of the fjord and the Gerlache Strait, and it was also found in trace amounts (<0.2% of diatom ASV abundances) at the innermost stations during both seasons. It had 99.3% nucleotide similarity to Chaetoceros calcitrans, a species so far unreported in Antarctic waters. Chaetoceros cells were also seen by microscopy, although further delineation was not possible based on morphological characteristics. _16S_ASV12 peaked in relative abundance at SB (4 April 2016) in the austral fall (Fig. 4A). This diatom was likely Odontella weissflogii, which was observed by light microscopy (36) (Fig. S5), but this species has no 16S sequence in the GenBank database. O. weissflogii is phylogenetically close to the odontelloid Triceratium dubium, based on other gene markers (37), and _16S_ASV12 had 98.2% nucleotide identity to T. dubium. Within the fjord during austral spring, the most abundant diatom ASV was _16S_ASV41 (Fig. 4C). Based on top BLASTn results, _16S_ASV41, as well as _16S_ASV17, _16S_ASV25, _16S_ASV74, _16S_ASV124, and _16S_ASV126, are all likely members of the Thalassiosirales. Many of the Thalassiosirales species are also unfortunately lacking representative 16S rRNA gene sequences in available databases. Several Thalassiosirales species could be observed by microscopy though, with species of Shionodiscus and Thalassiosira being notable (Fig. S5). In the austral fall, _16S_ASV48, which had 100% nucleotide identity to Fragilariopsis cylindrus, was present at all but stations SB (4 April 2016) and Outer Basin B (OBB) (19 April 2016) and was among the most abundant for diatoms. Diatoms overall showed a significant positive correlation with distance from the glacier and depth of the mixed layer, defined here as the region of the upper water column where surface wind-driven mixing results in homogenous temperature and other physical oceanographic characteristics (Fig. 4B).

10.1128/mBio.02973-21.5 FIG S5 Microscopy images showing a selection of diatoms identified, including Odontella weissflogii (a, b), Shionodiscus gracilis (var. gracilis) (c), Shionodiscus poroseriatus (d), Thalassiosira lentigiosa (e), Thalassiosira oliveriana (f), Thalassiosira cf. tumida (g), and Fragilariopsis cylindrus (h, i). Download FIG S5, TIF file, 2.6 MB. Copyright © 2021 Hamilton et al. 2021 Hamilton et al. https://creativecommons.org/licenses/by/4.0/ This content is distributed under the terms of the Creative Commons Attribution 4.0 International license.

The other major stramenopile taxa groups observed were bolidophytes, dictyochophytes, and pelagophytes, which at times comprised considerable percentages of plastid amplicons. For example, bolidophytes sometimes exceeded diatoms and consisted of 9 ASVs, most of which lacked high identity matches in GenBank, except for the second most abundant, _16S_ASV58, which had 99.6% identity to Triparma laevis. This taxon was reported previously in the WAP based on 18S rRNA genes (38) and was originally found in the North Pacific (39). Dictyochophytes consisted of 13 ASVs, with six being phylogenetically placed with Helicopedinella, three as sister to the Pseudopedinella and CCMP2098 clades *sensu* Choi et al. (26), and one each with Florenciella parvula, Florenciella clade II, and Dictyocha. Pelagophytes showed the lowest 16S-based diversity of all the stramenopiles, with three ASVs, placed with environmental clades PEC-I, PEC-V, and PEC-IX, respectively (26). Pelagophytes and dictyochophytes each exhibited a significant negative correlation between relative amplicon abundance and temperature (Fig. 4B), and the former was negatively correlated with day length. Additionally, pelagophyte, dictyochophyte, and bolidophyte amplicon abundances were each positively correlated with phosphate concentrations.

Cryptophytes exhibited higher relative abundances than haptophytes and other groups of phytoplankton discussed thus far, apart from the diatoms taken as a group (Fig. 4A). Haptophytes were dominated by _16S_ASV68 and _16S_ASV151, the first having 100% nucleotide identity to Phaeocystis antarctica and the second having 98% identity to Chrysochromulina parva (Table S2). Both had relatively low contributions, with the maximum being 6% of total plastid amplicons at Inner Basin B (IBB) during the fall (Fig. 4A). Moreover, haptophyte relative abundance was negatively correlated with temperature and day length (Fig. 4B). For cryptophytes, on average, 85.2 ± 10.0% (*n* = 25) of their 16S rRNA amplicons were formed by two sequence variants (_16S_ASV2 and _16S_ASV4) at each station, which differed from each other by two base pairs, while 7 other ASVs formed the rest of the cryptophyte community. An “uncultured marine cryptophyte clone” sequence from near the South Shetland Islands (59.3792 S, 55.7742 W; ∼700 km north of our sampling region) had the closest (99.6%) nucleotide identity to our dominant cryptophyte 16S ASV; however, the closest available 16S rRNA gene sequence from a cultured species was Teleaulax amphioxeia at 97.1% nucleotide identity. In austral spring, cryptophytes comprised up to 93% of the total plastid amplicons. In fall, when Chl *a* levels and overall phytoplankton abundances were low, cryptophytes formed up to 45% of plastid amplicons. For both seasons, cryptophyte relative abundance was negatively correlated with distance from the glacier (r = −0.42, *P* = 0.038) (Fig. 4B).

### A phylogenetically distinct cryptophyte dominates.

Our studies performed thus far established a covariance between diatoms, made up of many known taxa, and a dominant but seemingly unknown cryptophyte (_16S_ASV2). Like cryptophyte _16S_ASV2, the corresponding dominant _18S_ASV1 did not appear to come from a cultured cryptophyte. _18S_ASV1 did have 100% nucleotide identity to an Antarctic sequence from Ace Lake (GenBank accession number HQ111513), which came from a cryptophyte that was briefly in culture but then lost (40). The length of the amplicons generated here precluded phylogenetic analysis, apart from statistical placement of ASVs on curated reference trees. We therefore constructed 18S rRNA gene clone libraries, using DNA from a Gerlache Strait sample, and recovered full-length sequences that matched _18S_ASV1 at 100% nucleotide identity.

**FIG 5:**
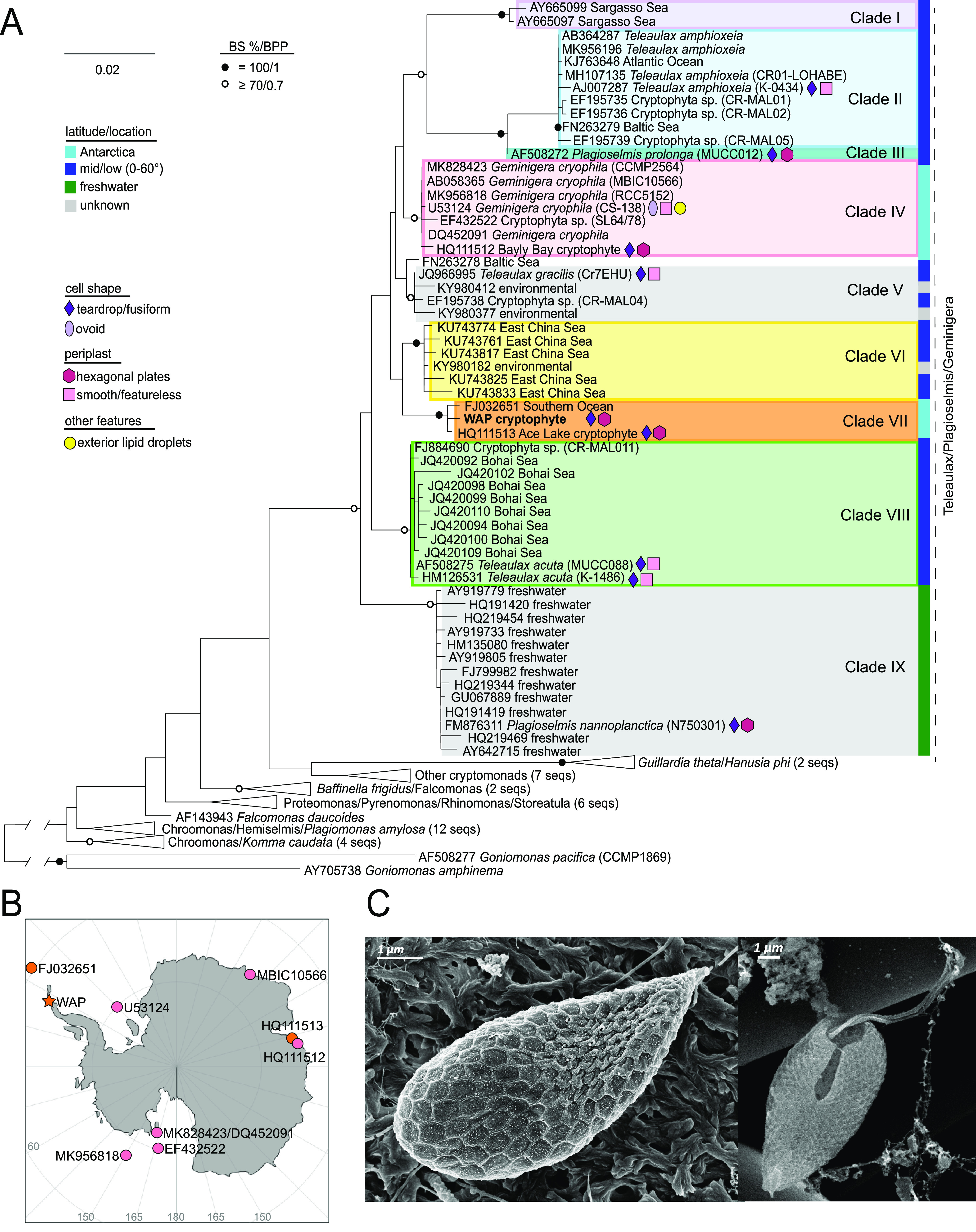
Phylogeny, morphology, and distribution of the dominant cryptophyte in this study. (A) Maximum-likelihood phylogeny of 18S rRNA genes from cryptophytes within the Teleaulax/Plagioselmis/Geminigera lineage, revealing the position of the WAP cryptophyte clone sequence (in bold). Taxon names are taken directly from GenBank and include strain identifiers and accession numbers. Environmental sequences are named by their location or as “environmental” when the location could not be determined. Symbols represent key morphological traits. The right-hand colored bar signifies the latitude at which the sequence-containing sample was taken (or the latitude of isolation for sequences from cultured representatives). Potential clades are highlighted and identified with a roman numeral. (B) Map of the Antarctic continent with stations where full-length cryptophyte sequences originated, either as cultured isolates or environmental samples. (C) Scanning electron microscopy (SEM) images of the WAP cryptophyte where the external morphological features can be appreciated.

The full-length sequence of the dominant cryptophyte in our study branched within the morphologically variable cryptophyte Teleaulax/Plagioselmis/Geminigera (TPG) lineage (41) (Fig. 5A). Although the innermost nodes (backbone) of the topology were generally unsupported, we were able to identify 9 clades within the TPG lineage that were moderately (>70/0.7) to fully supported by bootstrap values and posterior probabilities (Fig. 5A). All Antarctic sequences belonged to two statistically supported clades. Our WAP sequence formed a clade (here named clade VII) with the Ace Lake sequence, coming from the other side of Antarctica, and a Southern Ocean sequence from near the South Shetland Islands (GenBank accession number FJ032651; Fig. 5B). Sequences from the cultured Antarctic cryptophyte G. cryophila branched in clade IV, as did one from Bayly Bay (GenBank accession number HQ111512), near Ace Lake (40). Five other clades contained cultured taxa (II, III, V, VIII, and IX). Three were devoid of cultured representatives, namely, clade VII from our study; clade I, containing Sargasso Sea environmental clones; and clade VI, containing East China Sea environmental clones. Additionally, while sequence identities were often quite high or overlapping (Fig. S6), each clade had consistent, unique synapomorphies that differentiated it clearly from other clades (Fig. S7). Newly identified clade VII had a total of 8 unique nucleotide polymorphisms relative to clades IV and VI (across the entire gene sequence).

10.1128/mBio.02973-21.6 FIG S6 Sequence identity matrix of aligned Antarctic cryptophyte (clades IV and VII) 18S rRNA gene sequences. Download FIG S6, TIF file, 0.4 MB. Copyright © 2021 Hamilton et al. 2021 Hamilton et al. https://creativecommons.org/licenses/by/4.0/ This content is distributed under the terms of the Creative Commons Attribution 4.0 International license.

10.1128/mBio.02973-21.7 FIG S7 Subsection of the alignment of the 18S rRNA gene from cryptophytes representing the clades shown in the phylogenetic tree in Fig. 5a, revealing numerous clade-specific polymorphisms within the V9 region. Gaps represent positions present in other cryptophytes (in the full alignment) but absent from the subset shown. Download FIG S7, TIF file, 0.7 MB. Copyright © 2021 Hamilton et al. 2021 Hamilton et al. https://creativecommons.org/licenses/by/4.0/ This content is distributed under the terms of the Creative Commons Attribution 4.0 International license.

Based on the phylogenetic reconstruction, clade VII appeared to represent a novel species within the TPG lineage. We then paired the phylogenetic results with light and scanning electron microscopy studies. All cryptophytes observed in our samples (*n* = 128), with the exception of the samples from SB (20 December 2015), corresponded to a singular morphotype, which we therefore considered to be the WAP cryptophyte identified via molecular analysis and flow cytometry. This morphotype also matched descriptions of WAP cryptophytes observed previously (12). Light microscopy measurements indicated it was 11.3 ± 2.1 μm in length and 5.1 ± 0.6 μm in width (mean ± standard deviation of 41 individuals from both austral spring and fall). Morphologically, the novel cryptophyte differs from G. cryophila, which has a rounded or ovoid shape, a featureless periplast, and a characteristic warty aspect due to the accumulation of lipid droplets in the peripheral cytoplasm (27), as well as from Teleaulax species, which have a teardrop shape and featureless periplast (42). The novel cryptophyte has a teardrop shape, warty appearance, and hexagonal-to-rectangular plates that comprise the periplast (Fig. 5C). Furthermore, it has a conical tail with no plates and two flagella, slightly shorter than the body, emerging sideways from a ventral furrow. These characteristics aligned with physical traits attributed to Plagioselmis genus, yet the specifics of its morphology did not fit any currently described taxon (12). These results led us to conclude that clade VII captures members of an unknown cryptophyte species, that we refer to here as clade VII, to avoid naming prior to reevaluation of the entire TPG lineage.

### Cryptophyte clade distributions across the oceans expose niche differentiation.

**FIG 6:**
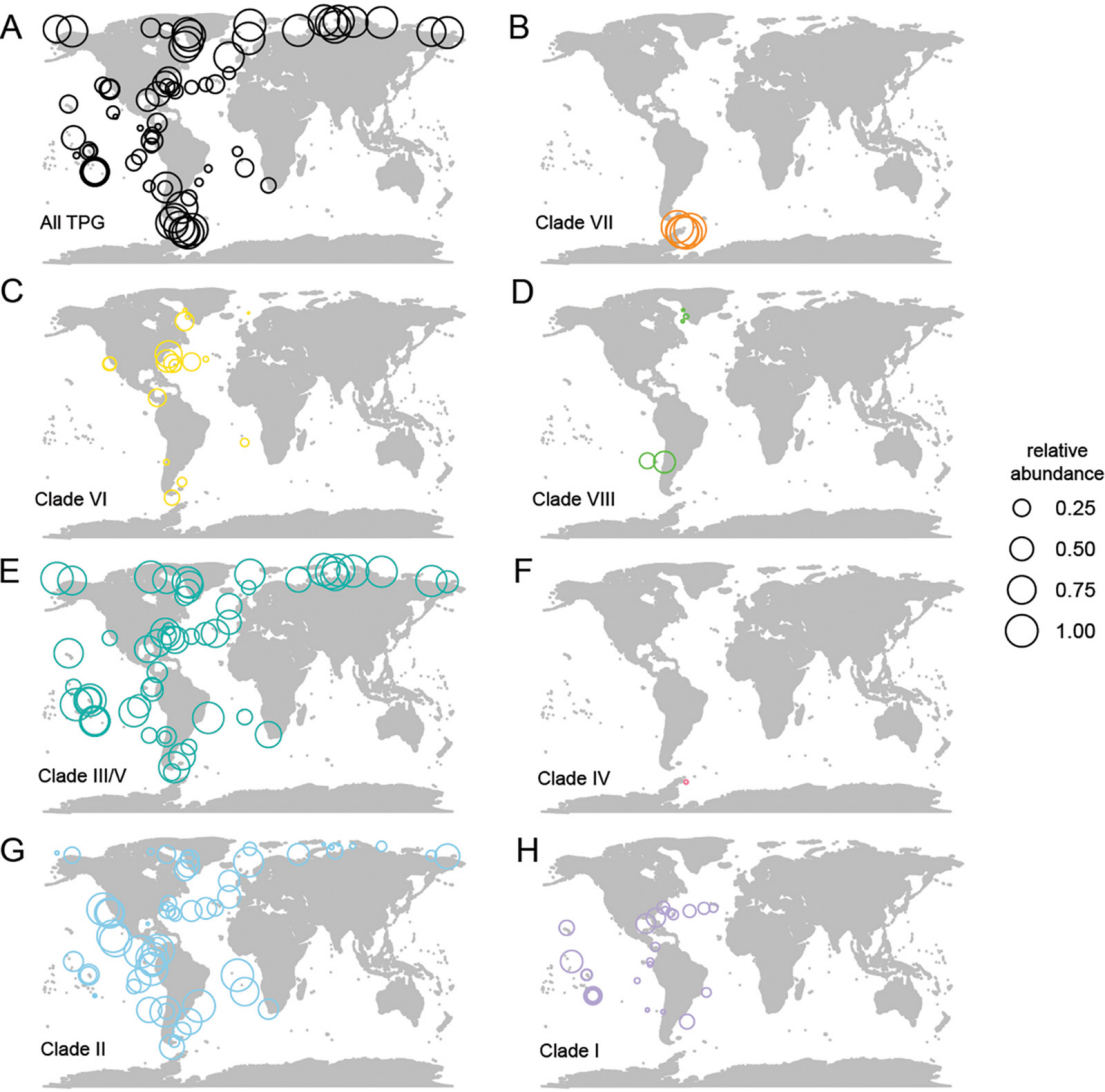
Global cryptophyte distributions in the surface ocean. (A to H) Maps of stations from the *Tara* Oceans expedition where surface seawater samples (>0.8-μm-filter-size fraction) were taken for V9 18S rRNA gene sequencing (data from Ibarbalz et al. [43]). Rings are sized to represent relative amplicon abundance. The first map (A) shows the relative abundance of all TPG clade cryptophyte amplicons out of the total cryptophyte amplicons in each sample. The following maps show the relative abundance of amplicons representing clade VII (B), clade VI (C), clade VIII (*Teleaulax acuta*) (D), clades III/V (*Plagioselmis prolonga/Teleaulax gracilis*) (E), clade IV (Geminigera cryophila) (F), clade II (*Teleaulax amphioxeia*) (G), and clade I (H) out of the total TPG clade cryptophytes. Classifications were based on the phylogenetic reconstruction of cryptophytes using the full-length 18S rRNA gene (see Fig. 5a) and the TPG clades designated within. Specific polymorphisms in the V9 region that connected to each clade were identified manually, which enabled the designation of representative sequences and their corresponding ASV within the *Tara* Oceans data set for each clade.

While the overall topology of the 18S rRNA gene phylogenetic reconstruction was not well supported, many clades had support, as well as clade-specific synapomorphies, especially in the 18S V9 region. While, like most clades, clades III and V differentiated from all other clades within the V9, they could not be distinguished from each other (Fig. S8). Each clade (Fig. 5A) featured 100% nucleotide identity within itself for the V9 region, with the exception of environmental clade VI (East China Sea) and the freshwater clade (IX), where there were three variants per clade that differed by 1 nucleotide (nt) each (Fig. S7). We exploited the clade-identifying synapomorphies to examine TPG distributions in *Tara* Oceans V9 18S amplicon data (43). The results show that cryptophytes are broadly distributed from pole to pole, as supported by prior studies on different ocean regions (41). Furthermore, our analyses showed that the TPG are the dominant marine cryptophyte lineage throughout the global ocean (Fig. 6A).

Our more specific synapomorphy-based analyses provided unprecedented insight into the molecular diversity of TPG cryptophytes and the environments they inhabit. Members of clade II (Teleaulax amphioxeia) and clades III/V (Plagioselmis prolonga and Teleaulax gracilis) were present in temperate and tropical environments, as well as at high Arctic latitudes, but were not detected in the WAP and Southern Ocean (Fig. 6E and G). Moreover, although they were frequently detected together, this was not always the case. The newly discovered clade VII is limited to high southern latitudes. To test whether it might be present in freshwater environments, where cryptophytes can be important (44), we also queried a publicly available riverine data set (45). Although a definitive comparison could not be made due to the clustering methods used to generate operational taxonomic units (OTUs) in Arroyo et al. (45), we did not detect any OTU with >98% nucleotide identity to clade VII. We did find that clade VII dominates in the region of Antarctica (WAP) sampled by *Tara* Oceans (Fig. 6B). Members of clade IV (G. cryophila) were found at just one WAP station, although they were present in multiple samples of our study. At the *Tara* Oceans WAP sites, clade IV had much lower contributions than those of clade VII to TPG relative abundance. Finally, we found that, like our V9 _18S_ASV1, the dominant cryptophyte V4 18S ASV identified in a recent WAP study (19) had 100% nucleotide identity (to the V4 region) of our clade VII full-length sequence.

## DISCUSSION

Studies examining protistan diversity using high-throughput 18S rRNA or 16S rRNA (for phytoplankton only) sequencing in polar regions have been performed across the Arctic using survey sampling (e.g., references 43, 46, 47), but challenges in accessing Southern Ocean and Antarctic fjords and coastal zones have resulted in more restricted knowledge of Antarctic communities (48–50). Open-water periods in polar fjords tend to be productive and sensitive to alterations in the cryosphere-ocean interface. This sensitivity, as well as the fact that they provide habitat for foraging by many marine animals, call for focused, repeated studies of primary producers in these ecosystems in order to examine their responses to environmental conditions. Although Arctic fjords have been well studied (51, 52), they differ from Antarctic fjords because they receive strong annual glacial meltwater inputs, providing cold freshwater and terrigenous sediments, which leads to a significant down-fjord gradient in both pelagic and benthic habitats (53). In contrast, glacial meltwater influences are milder in Antarctic fjords, such that nutrients are still injected and some stratification occurs, but without the sediment loading and high turbidity associated with stronger meltwater inputs. The extent of meltwater input is highly sensitive to climate change (54), a factor that motivated this research and our study design for investigating extant communities in Antarctic fjords during open-water periods. Through this high-resolution examination of fjord protistan molecular diversity, alongside quantitative and imaging approaches, we were able to shed light on varied differentiation patterns between phytoplankton groups, including an evolutionarily distinct, uncultivated lineage of cryptophytes of importance within a fjord and in the greater WAP.

### Protistan communities in polar environments.

The major protistan groups seen here (Fig. 2) are also present in Arctic fjords (51, 55), and a number have been reported in WAP studies. Our study location was ∼60 km from a region sampled near Anvers Island (14), a distinct environment from fjords that has similarities to the Gerlache Strait. Our location was intermediate to the coastal South Shetland Islands (15, 18, 49) and Marguerite Bay (16), a true bay (not a fjord). The sequence analysis resolution for the two studies of Southern Ocean waters near the South Shetland Islands was most similar to ours, although they used the V4 18S region, not V9. The more “mysterious” and small-sized heterotrophic microbial eukaryotes, also present in Arctic fjords and seas (47, 51), are Picozoa, telonemids, and MASTs. While the ecosystem roles of uncultured MAST lineages are unclear, several are known predatory heterotrophic nanoflagellates, including MAST-1, MAST-2, MAST-7, and MAST-8 (56, 57). The community composition here was similar to that in the most comparable South Shetland Island study (49); however, overall diversity in the previous study was less than that in ours, likely due to their use of 97% nucleotide identity OTUs, which would minimize diversity estimates.

The results suggest that potentially predatory dinoflagellate taxa may be common, based on 18S ASV relative abundances (Fig. 2B), corresponding to results from the South Shetland Islands (49). The dinoflagellates were highly diverse and confirmed early hypotheses, based on more limited 18S rRNA gene sequencing, that there are small, still undescribed dinoflagellates in the Southern Ocean (5, 58, 59). We observed ASVs affiliated with potentially kleptoplastidic Gymnodiniales, which acquire and use plastids from other phytoplankton and also operate as heterotrophs through seasonal periods of complete darkness (47, 60). Although relative abundances did not always correspond, at a general level, the phytoplanktonic taxa identified via 18S and 16S ASVs (Fig. 2 and 4) were equivalent, providing a backdrop against which we could embed resolved taxonomic analyses using 16S ASVs. The 16S rRNA gene approach has been reported to provide improved insights into organismal relative abundances, due to the large variation in 18S rRNA gene copy numbers among eukaryotes and the oftentimes low phylogenetic resolution of the V9 18S rRNA region (25, 26, 61).

### Molecular diversity and identities of Antarctica’s “small flagellated” phytoplankton.

“Small flagellates” or “mixed flagellates” have been noted repeatedly as important contributors to WAP phytoplankton communities (7, 9, 21, 23). This group is a conglomerate of multiple phytoplankton taxa (i.e., pigmented), many of which are not further identifiable by microscopy or HPLC. Here, we showed that the “mixed flagellates” of Andvord Bay (e.g., reference 23) include prasinophytes, bolidophytes, pelagophytes, dictyochophytes, and haptophytes. These small flagellates comprised 38% of the total chlorophyll *a* (Chl *a*) based on averaged values from austral spring stations in Andvord Bay (21). They are also the second most abundant phytoplankton group based on microscopy counts in austral spring, after unidentified cryptophytes (23). Within the “other phytoplankton” or “unidentified or mixed flagellates” categories, we surmise that several taxa are important. For example, we observed Phaeocystis antarctica in both 18S and 16S rRNA gene amplicon analyses, and it has been reported elsewhere in the WAP during austral spring (62). Other small flagellates may have been misassigned to diatoms (or to a broad category of “other phytoplankton”) when HPLC is used. Both bolidophytes and dictyochophytes have pigments that overlap those used as diatom markers, such that HPLC analyses would classify them as diatoms (26). This misidentification may have important biogeochemical implications because these lineages lack diatom-like frustules. Bolidophytes have either siliceous plates or are unsilicified (38, 63), and dictyochophytes have variations with respect to siliceous structures or the lack thereof. Importantly, a number of these lineages have members with roles extending beyond classification as “simple” primary producers. The haptophyte P. antarctica is a plastid source for some potentially kleptoplastidic Antarctic dinoflagellates (64) that were present in our data. Moreover, other haptophytes, as well as dictyochophytes, contain some predatory photosynthetic mixotrophs. These complexities alter the environmental constraints and selective processes that act upon them compared to those that act upon purely photosynthetic taxa (65).

The prasinophytes observed would also likely fall into the “other, mixed, or unidentified” phytoplankton categories of prior Antarctic studies. The dominant pyramimonad 18S ASV had 100% nucleotide identity to Pyramimonas gelidicola, a species isolated from Antarctic waters (66, 67). Both our 18S and 16S data sets contained M. polaris and Bathycoccus prasinos ASVs. These two taxa have been reported in the South Shetland Islands, Marguerite Bay (16, 18), the Amundsen Sea, and circumpolar waters surrounding Antarctica (68, 69). In Fildes Bay (South Shetland Islands) *Micromonas* had high relative abundances, comprising up to 47% of the total V4 18S amplicons, and a possible new ASV variant (in addition to *M. polaris*) was reported (18). This differs from our observation of two *M. polaris* 18S V9 ASVs (100 and 99% nucleotide identity to the cultured strain); however, the V4 region is known to resolve some prasinophyte groups better than the V9 region (61). Additionally, we found that *M. polaris* was consistently present, but at low relative abundances, in both 16S and 18S amplicon data, as confirmed by qPCR (see Fig. S4 in the supplemental material). Collectively, these studies point to unknowns regarding environmental drivers behind species proliferation in specific Antarctic regions or seasons. Moreover, they highlight some specific small flagellates as having bipolar importance, particularly *M. polaris* (69), which was once thought to be endemic to the Arctic.

### Phytoplankton community structure and links to environmental parameters.

A factor thought to contribute to productivity and aggregations of large mammals in Andvord Bay, is that it is “dynamically quiet,” lacking the strong wind forcing of the ambient ocean and facilitating some level of stratification. We observed a significant positive correlation between the depth of the mixed layer and relative diatom amplicon abundance, an indication of requirements for higher nutrient concentrations than those available when the water column is more strongly stratified (Fig. 3B). Otherwise, the macronutrient that correlated most strongly with multiple phytoplankton groups was phosphate. Bolidophytes, dictyochophytes, pelagophytes, and pyramimonads all exhibited positive correlations with increased phosphate concentrations (Fig. 4B). In another analysis of Andvord Bay (21), no relationship was seen between “mixed flagellates” and these parameters, likely because distinct lineages were merged, highlighting the importance of resolving phytoplankton taxa at a resolution that connects with niche partitioning.

While macronutrient availability is an important factor shaping phytoplankton communities in many regions of the global ocean, in polar fjords, glacial dynamics are also extremely important. Distance from the glacier exhibited the strongest relationships with the relative amplicon abundances of different phytoplankton groups. Specifically, distance from the glacier terminus was positively related to relative mamiellophyte and diatom amplicon abundances (Fig. 4B). In contrast, cryptophyte amplicon relative abundances were negatively correlated with distance from the glacier, and their cell abundances were also generally highest in the inner part of the fjord (Fig. 3 and 4). This suggests that the glacier plays an important role in shaping the phytoplankton community in Andvord Bay, despite its characterization as a “cold” fjord with relatively little influence from glacial meltwater in terms of physical oceanography (70). While glacial meltwater was statistically linked to total phytoplankton cell abundance based on flow cytometry measurements, the specifics of this relationship are unclear, particularly when Bonferroni correction methods are applied. Corroborating the findings of Pan et al. based on HPLC data (21), our cryptophyte cell counts were positively correlated with temperature. Importantly, this points to the potential for cryptophyte proliferation being enhanced in this region due to future climate warming.

As discussed, diatoms are important stramenopiles in Andvord Bay, and elsewhere in both Arctic fjords and Antarctic environments. However, diatoms are diverse (71), and our data show that patterns arise at the 16S ASV level that are obscured at the group level. For example, F. cylindrus (_16S_ASV48), a well-recognized member of the WAP phytoplankton community (13), was present at low relative abundance at all spring stations within the fjord and Gerlache Strait, but dominated diatom amplicons at SB (20 December 2015) (Fig. 4C). In fall, the opposite occurred, where this taxon occurred at higher relative amplicon abundances within the fjord and strait. One explanation of taxon-specific trends revealed by our molecular data set could be that there is a seasonal pattern of an initial austral spring presence of *F. cylindrus* out on the shelf, with a transition toward the nearshore environment, including within the fjords, during the fall. Other diatom ASVs showed differing patterns, emphasizing the need for high taxonomic resolution.

A unique feature of polar fjords is that there are occasional katabatic wind events, driven by strong winds originating on the continental ice sheet (70). Our sampling included 3 days at Sill 3 that spanned an austral spring katabatic wind event (70). Prior to the event (29 November and 8 December 2015), cryptophyte amplicons dominated plastid sequences (Fig. 4A), and cryptophyte cells were abundant. After the event (17 December 2015), relative cryptophyte amplicon frequency was lower, and diatoms dominated amplicons. The strong down-fjord winds induced wind-activated mixing that replenished photic zone nutrients (70), with nitrate higher toward the end of the event (13 December 2015) and drawdown observed by 17 December 2015 (72). Thus, katabatic wind events appear to have a role in resetting phytoplankton communities and in the relative contributions of different lineages in Antarctic fjords, adding a consideration beyond the oft-discussed influences of ice retreat and the degree of temperature-induced surface water stratification.

### Molecular insights into the novel clade VII WAP cryptophyte and overall TPG lineage.

While cryptophytes have been observed as one of the key phytoplankton taxa in the WAP by a multitude of studies (e.g., references 9, 10), almost nothing was known about their molecular diversity. Geminigera cryophila is the only Antarctic cryptophyte in culture, and hence the only species where molecular sequence data have been connected with morphological information. Indeed, to date, phylogenetic analyses point to a disconnect between evolutionary analyses and morphologically defined genera within the entire TPG lineage (Fig. 5A). One explanation has been that life history-dependent dimorphisms like those reported in the non-TPG genus Cryptomonas (42) complicate interpretation. *Plagioselmis prolonga* and *Teleaulax amphioxeia* have been proposed to be the same genus and species, with P. prolonga representing the haploid state and T. amphioxeia the diploid state, primarily based on morphological changes within cultures (73). Unfortunately, several TPG lineages recovered here are not represented in culture, precluding further scrutiny. Furthermore, resolution of marker sequences used, possible erroneous naming in GenBank, and low-quality marker gene sequences for some reference taxa have complicated understanding of the TPG. Here, both phylogenetic analyses and conserved V9 18S polymorphisms distinguished *P. prolonga* and *T. amphioxeia*, indicating they are not the same species.

Environmental studies have also presented inconsistencies. For example, the Ace Lake (GenBank accession number HQ111513, clade VII) and Bayly Bay (accession number HQ111512, clade IV) cryptophytes are proposed cryptomorphs of G. cryophila (40). The suggested dimorphism is a warty, ovoid cell type (campylomorph form) seen in aged cell cultures, for which microscopy images are lacking, and it is unclear if this was observed for both the Ace Lake and Bayly Bay cultures (both now lost). Moreover, dimorphism has not been observed in available cultures of G. cryophila. Here, placement in separate bootstrap-supported clades (Fig. 5) and conserved polymorphisms observed between the V9 sequences of clade IV and clade VII (Fig. S7) make cryptomorphy seem unlikely between the Ace Lake strain and G. cryophila. Although our analyses facilitated reinterpretation of TPG inconsistencies, an overall reevaluation of genus-level differentiation would be exceptionally helpful and would benefit from targeted PCR and genome sequencing efforts paired with cell imaging, if not cultures.

The multifaceted approaches underpinning our study provided a platform for bringing together results from recent reports on uncultured WAP cryptophytes. Our findings, based on integrated flow cytometry, microscopy, amplicon sequencing of two molecular markers, and full-length 18S rRNA gene sequencing, demonstrate that the dominant cryptophyte belongs to a unique lineage, here named TPG clade VII. It is both phylogenetically and morphologically distinct from G. cryophila (clade IV; Fig. 5). We observe that the clade VII cryptophyte and G. cryophila are consistently found in our samples at ratios greater than 4:1. Two recent studies, one involving microscopy (12) and the other V4 18S rRNA amplicon sequencing (19), also found that G. cryophila may not be the primary WAP cryptophyte species. The V4 18S ASV study did not sample fjords but rather focused on repeat transect sampling in WAP sites during summer and reported that a cryptophyte ASV that differed in sequence from that of G. cryophila had higher relative abundance than G. cryophila. We were able to compare the identified ASV and the V4 region of our full-length clade VII gene sequence and found they had 100% nucleotide identity. Hence, taking into account our integrative results and indications from prior studies of Ace Lake (40) and those of the nonfjord WAP transects (19), the novel TPG clade VII appears to be an important cryptophyte in multiple Antarctic regions, particularly in the WAP.

### Linking high-resolution taxonomy to local and global spatial community structure.

Our analyses identified a key phytoplankter in a more taxonomically and evolutionarily relevant way and established important environmental linkages. Full-length sequencing alongside identification of evolutionarily conserved polymorphisms between TPG clades within the V9 18S region allowed us to place cryptophyte diversity in a broader context using *Tara* Oceans V9 data (43). Globally, TPG group members appear to dominate over other cryptophytes (Fig. 6A). Some cryptophyte clades were broadly distributed in tropical and temperate waters (clade I; Fig. 6H) or extended into subpolar Arctic waters as high as 69° N (clades VI and VIII; Fig. 6C and D). Overall, the cryptophytes seen in the Arctic were also detected in tropical and temperate waters extending to 60° S (clades II and III/V), but were not detected in Antarctic waters (Fig. 6E and G). In contrast, we found no evidence for cryptophytes in either clade VII or cade VI (the cultured G. cryophila) being present in Arctic fjords, in other Arctic waters, or in the temperate and tropical regions sampled (Fig. 6B and F). Thus, it appears that the newly discovered clade VII is endemic to Antarctica, as is clade IV. Moreover, clade VII has much higher relative abundances than G. cryophila in our studies, as well as in *Tara* Oceans cryptophyte data from the Southern Ocean (Fig. 5 and 6).

With respect to the broader phytoplankton community, we also observed that when the absolute abundance of noncryptophyte phytoplankton increased, so did the abundance of the seemingly Antarctic-specialized cryptophytes in spring months (*r* = 0.75, *P* = 2.2 × 10^−16^), as did the relative proportion of cryptophytes (*r* = 0.93, *P* = 3.8 × 10^−31^). This indicates that the phytoplankton community as a whole responded positively to certain environmental cues but that the cryptophyte response (in terms of cell numbers) was more pronounced. The maximum cryptophyte abundances were between 3,000 to 4,000 cells · mL^−1^, similar to results of another WAP study (74). Observed cryptophyte abundances were within range of those for most blooms reported by microscopy (compiled in reference 12), although blooms reaching 1.6 × 10^4^ cells · mL^−1^ have also been reported near Anvers Island (8).

What can these findings tell us about the oscillation between cryptophytes and diatoms observed in our amplicon data or reported previously in the Antarctic using other methods (75)? Although not all studies employed statistical analyses, and none identified the cryptophyte(s) or diatoms involved, speculations regarding Antarctica’s diatom-cryptophyte dichotomy include cryptophyte preference for lowered salinity (9) and/or increased temperature (13, 21) or, conflictingly, increases in response to low-salinity, colder waters (75). We find that (i) WAP communities contrast sharply in that cryptophytes have a single taxonomic dominant, while diatoms are diverse and the relative abundances of specific diatoms shift over time; (ii) the cryptophyte present appears to be endemic to the Antarctic and potentially has more limited opportunities for dispersal beyond the Southern Ocean, whereas the diatoms present have much larger distributions and may have arisen elsewhere; and (iii) clade VII cryptophyte abundance is strongly correlated with increased seawater temperature, a factor that is predicted to be enhanced by climate change, whereas the diatoms showed a significant relationship with seasonally reduced stratification, potentially reflecting greater nutrient requirements. Additionally, the katabatic wind event captured in our study showed that diatoms responded to reduced stratification and increased nutrients over the course of days. Thus, over both seasons and shorter time scales, cryptophytes and diatoms responded to different environmental factors, with cryptophyte abundances being most strongly correlated with the impacts of warming.

Finally, why are the overall phytoplankton group patterns so different in Antarctic fjords compared to those in the Arctic? In the Arctic, diatoms appear to be the main bloom-forming taxa, with blooms typically seen in the spring in either the inner (76) or outer fjord (52). While cryptophytes have also been reported in many Arctic fjords, other algal groups, including the prymnesiophytes and photosynthetic dinoflagellates, have been noted as more important components of the phytoplankton community when diatoms are lower in abundance (76, 77). Our results point to dominance of the clade VII cryptophyte in the WAP and fjords within and suggest its great importance to blooms based on HPLC and microscopy studies in the same region (12, 21). One potential consideration is that the cryptophytes seen in the Arctic appear to be generalists also found in temperate and tropical waters. In contrast, clade VII so far has not been observed elsewhere in the ocean, and its dominance over clade VII may be connected to its ability to proliferate in the WAP and its fjords. Diatoms are less abundant at these times, which again may connect to different environmental drivers or to the fact that most are bipolar, and some even more generalist in their distributions. Future studies examining differences in phytoplankton community structure between poles would benefit from the inclusion of niche specialization and dispersal processes (69, 78) as potential contributing factors.

### Summary.

Knowledge of the extant diversity in Antarctica and its “hot spot” fjords is a priority. Here, we elucidated protistan diversity by combining results from two expeditions designed to examine seasonal differences in open-water primary producers in an Antarctic fjord noted for high productivity and diverse megafauna. Our studies reveal variations in how the diversity of different phytoplankton lineages manifests in this important polar region, and its connections to climate-sensitive seasonal perturbations. In identifying the hitherto “unidentified small flagellates” that are important components of the phytoplankton community in Antarctica, we were able to single out taxa within this group that have bipolar distributions, such as the diatom *Fragilariopsis cylindrus* and the prasinophyte *Micromonas polaris*. Some phytoplankton groups as a whole exhibit high diversity in Antarctica, such as diatoms, indicating extensive niche differentiation, while others exhibit narrow diversity and extreme dominance by a single taxon, like the clade VII cryptophytes. Indeed, the phylogenetically distinct cryptophyte lineage elucidated here had been noted as a potentially different lineage or morphotype in prior studies of Antarctic waters (12) but without supporting evolutionary data. By incorporating our results with those of other studies, we found a clade VII member to be the dominant cryptophyte in the fjord, outside the fjord, across the broader WAP, and at other Southern Ocean sites, almost to the exclusion of other cryptophytes. Alongside clade VII results, our analyses indicate that trace, consistent contributions are present from the known, cultured Antarctic cryptophyte G. cryophila. Unfortunately, the novel clade VII cryptophytes have not yet been cultured and appear to be endemic to Antarctica, making access restricted. Importantly, the abundance of the clade VII cryptophyte was positively correlated with increased water temperature. While WAP fjords are considered to be “cold,” with weaker meltwater influence relative to that in Arctic fjords, they are not immune to the effects of climate-induced warming. The strategies reflected in the dominance of a single endemic cryptophyte under conditions known to be enhanced by climate change and differing from periods characterized by a diverse diatom assemblage with bipolar representatives may point to future selection processes, with as yet unknown impacts on the larger food web structure.

## MATERIALS AND METHODS

### Environmental sampling.

Cruises to Andvord Bay, Gerlache Strait, and a western Antarctic shelf station were performed from 27 November 20 December 2015 (LMG 15-10) and 4 to 26 April 2016 (NBP 16-03). Niskin bottles for water collection were deployed on a rosette system with dual sensors measuring depth, temperature, salinity, fluorescence, and light transmission. Microscopy sampling was according to surface light level percentages (50%, 12%, and 1%), with 3% (final concentration) Lugol’s fixation. Flow cytometry samples were preserved with 0.25% electron microscopy (EM) grade glutaraldehyde (final concentration) (79). DNA samples were filtered onto 0.2-μm pore size and 47-mm diameter Supor filters under low vacuum and frozen at −80°C alongside flow cytometry samples. Methods for nutrients, Chl *a*, meltwater fraction, mixed layer depth measurements, and the calculation of distance from the glacier were described previously (21). Data on ice cover during the period of this study are available in Lundesgaard et al. (70).

### DNA extraction and amplicon sequencing.

DNA was extracted using a modified protocol of the Qiagen DNeasy plant kit (80), quantified using the QuBit double-stranded DNA (dsDNA) high-sensitivity assay (Life Technologies, Grand Island, NY). For amplicon sequencing, DNA was diluted with Tris-EDTA (TE; pH 8) to 1 ng · μL^−1^, with V1-V2 16S rRNA amplicon sequencing and PCRs performed as described previously (34). Each reaction mixture included 5 ng template, 5 μL 10× buffer, 1 U Hi-Fi Taq, 1.6 μL 50 mM MgSO_4_ (Life Technologies), and 200 nM (each) forward primer 27F_ill 5′-TCGTCGGCAGCGTCAGATGTGTATAAGAGACAGagrgttygatymtggctcag‐3′ and reverse primer 338RPL_ill 5′‐GTCTCGTGGGCTCGGAGATGTGTATAAGAGACAGgcwgccwcccgtaggwgt‐3′; capital letters represent Illumina linker sequences on the 27F/338R primer pair (81). Reactions were cycled for 2 min at 94°C, 15 sec at 94°C (30 times), 30 sec at 55°C, 1 min at 68°C, and 7 min 68°C (for elongation). Purification was done using the MinElute kit (Qiagen, Valencia, CA), and product presence and removal of primer-dimers were verified by electrophoresis. V9 18S rRNA amplicons were generated as described above using the 1389F/1510R primer pair (24). Samples were sequenced using Illumina MiSeq v3 chemistry.

### 18S rRNA ASV generation and analyses.

18S ASVs were generated by trimming the raw amplicon sequences of primers using cutadapt v3.2 and inputting them into the DADA2 pipeline (v1.19). In brief, the steps included filtering and trimming reads, dereplication, paired read merging, and chimera removal. The identity of subsequent ASVs was determined via the classifier tool implemented in Qiime 2 (82). For an additional broad overview of the spread of eukaryotic diversity, see Wideman et al. (29). Briefly, V9 sequences were phylogenetically mapped onto a previously published 18S rRNA gene maximum-likelihood (ML) reference phylogenetic reconstruction that used a selected subset of sequences available in the PR^2^ database v.4.454 (83); short sequences (<400 bp) had been removed, as well those not spanning the V9 region and those of metazoans. Sequence redundancy was also been limited by retaining only representative sequences after clustering, such that the final alignment contained 20,939 18S rRNA gene sequences. The ML reference reconstruction was built using RAxML v.8.2 (84) under the GTR model with CAT approximation (29). A total of 885 V9 ASVs (representing 1,256,577 amplicon sequences) were then aligned against the unmasked reference tree sequences using MAFFT (85). Aligned V9 ASV sequences were then placed onto the reference ML reconstruction using EPA-ng v0.3.6 (86), which employs an RAxML evolutionary placement algorithm, under the GTR-CAT model. Subsequent to phylogenetic mapping, taxa with long branches were removed for display purposes if the terminal branch was longer than 3 substitutions per site, using information from the Newick utilities package (87), and the tree was rendered using the R package *ggtree* (88). Note that no V9 ASVs from our study mapped to these long branches.

### Phytoplankton cell enumeration.

Flow cytometry samples were analyzed on an Influx flow cytometer (BD, San Jose, CA) as described previously (79), using Fluoresbrite YG 0.75-μm beads as standards. WinList 7.0 (Verity Software House, Topsham, ME) was used to analyze listmode data files. Cyanobacterial populations were not identified, including Synechococcus. Cryptophytes were distinguished from other phytoplankton based on forward-angle light scatter (FALS) and phycoerythrin-derived orange fluorescence. For cell counts and dimension measurements, samples were observed at maximum amplification (400×) using an inverted optical light-emitting diode (LED) microscope (DM IL; Leica). For scanning electron microscopy (SEM), sample aliquots were filtered onto 0.2-μm polyamide filters and dehydrated through an ethanol dilution series (25%, 50%, 75%, and 100%) with final critical-point dehydration. Specimens were sputter-coated with gold-palladium and examined using Jeol JSM-6360LV and Zeiss Supra 40 instruments. Cryptophyte biomass was estimated by approximating average cell volume based on previous models (89) and the average cryptophyte size measured here. Cellular carbon content was estimated using a carbon:volume ratio for cryptophytes (90). Estimates of noncryptophyte phytoplankton biomass was generated by approximating average cell volume, assuming a spherical cell shape and 5.5 μm average length, and 237 fg C · μm^−3^ as the carbon:volume ratio (91).

qPCR and standard curves were performed with a *Micromonas* primer-probe set, MicroGen08 (MicGen08F, TGTTCAAAGCGGGCTTA; MicGen08R, ATGCCCCCAACTGTTCCTCTTAA; MicGen08P, 6-carboxyfluorescein [FAM]-CCATGCTGAAATATTCAAG-MGBNFQ), according to previously described methods (80) using an Applied Biosystems real-time PCR system (7500; Foster City, CA). Cycling conditions were 10 min at 95°C, followed by 45 cycles at 95°C for 15 sec and 60°C for 1 min. Inhibition tests with dilutions between 1:4 and 1:4,000 established 1:40 as being appropriate for templates. Assay detection limits were 10 template copies · well^−1^. Ribosomal DNA (rDNA) copies · mL^−1^ were calculated by accounting for the volume of seawater filtered, template added, and dilution factor used.

### Plastid ASV generation and analyses.

For V1-V2 16S rRNA amplicons, low-quality merged sequences and primers (at 100% match) were removed using cutadapt v1.16. A 10% read length window with a Q25 running-quality threshold was implemented for trimming low-quality bases. These data were input to the DADA2 pipeline (v1.14), and paired-end sequences were merged with a 20-nt overlap and no mismatches. The resulting ASVs were run through PhyloAssigner to determine phylogenetic classification (26). The taxonomic identity of select ASVs was also examined using BLASTn searches against the GenBank nr database.

### Cryptophyte full-length 18S rRNA gene sequencing and phylogenetic analyses.

To obtain full-length sequences, clone libraries were generated from 14 April 2016 Gerlache Strait DNA using a universal eukaryotic 18S rRNA gene primer set (92) as described previously (79). After cloning and purification, sequencing was performed using BigDye Terminator chemistry on an AB3730xl sequencer (Applied Biosystems), with initial screening using the 502F primer for the taxon of interest, and then with plasmid-targeted M13F and M13R primers. GeneStudio v2.2.0.0 was used to manually curate the assemblies.

The cryptophyte 18S rRNA gene sequence generated was used as a BLASTn (93) query against the GenBank nr database to retrieve sequences from related cultured taxa and from environmental sequences, in independent searches, that were then combined with sequences from prior cryptophyte reconstructions (42, 94). For multiple identical sequences, we performed some subsampling and, after preliminary alignment and tree building, further curated to remove sequences that had multiple unresolved bases, indicating poor sequence quality, or that were much shorter than the bulk of nearly full-length sequences. The resulting 93 representative sequences were aligned using MAFFT v.7.271 (85), using the “-auto option,” and ambiguous positions were removed using trimAl v.1.2 (“-gt 0.3, -st 0.001”) (95). In order to retain sequence FJ032651, which was one of the closest to our full-length cryptophyte sequence, we reduced the number of 5′ positions included (for the entire alignment), after observing that tree building with “missing positions” settings was not appropriately representing branch lengths. The final alignment consisted of 1,649 positions. The tree was inferred using RAxML v.8.2.9 (“-m GTRGAMMAI”) (96), and branch support was assessed with 1,000 nonparametric bootstrap replicates. Additionally, a Bayesian inference (BI) phylogenetic reconstruction analysis was performed with MrBayes (97), using the same model of evolution with two independent runs of 2,500,000 generations with four chains each (i.e., one cold and three heated) and sampling every 250 generations. After a burn-in of the first 25% of trees, posterior probabilities for node supports were computed.

To investigate TPG cryptophyte distributions, relative abundances of ASVs from this lineage were determined for *Tara* Oceans surface samples filtered onto a >0.8-μm size fraction (for consistency, this required omitting some stations) (43). A representative sequence database for each statistically supported TPG clade was created and used as BLASTn queries against all ASVs identified as “Cryptophyta” (43) using PR^2^. ASVs with 100% identity to a representative sequence for each TPG clade were identified and confirmed via alignment to the representative full-length sequence and a manual inspection. Their relative abundance computed within the total “Cryptophyta” amplicons; those with <10 reads in a sample were considered “not detected” for the respective station. To determine the possible presence of clade VII cryptophytes in a freshwater system, the V8-V9 region of the WAP cryptophyte 18S rRNA gene was used as a BLASTn query against OTU sequences labeled as “Cryptophyta” using both PR^2^ and SILVA119 in a study with samples from Middle Paraná River, Argentina (45). Other publicly available searchable data sets from freshwater metagenomics studies could not be searched due to the issues with assembling reliable 18S rRNA gene sequences via traditional metagenomics approaches.

### Statistical analyses.

For comparisons between flow cytometry-based cell abundances and environmental parameters, Bonferroni-adjusted Pearson correlations were calculated with the R package *stats* (v4.0.2). The R package *Hmisc* (v.4.1.1) was used to calculate Pearson correlations involving the relative 16S rRNA gene amplicon abundance of taxonomic groups, and this was visualized via the package *corrplot* (v.0.84). Rarefaction curves were created and diversity metrics calculated (Shannon index and species richness) using the R package *vegan* (v2.5-6) for the ASVs generated from the 18S and 16S (plastid only) data sets.

### Data availability.

The amplicon sequencing data set generated for this study can be found in GenBank under accession numbers SAMN21839445 to SAMN21839469. The clade VII cryptophyte sequence can be found in GenBank under accession number OK285281.

## References

[1] Turner J, Colwell SR, Marshall GJ, Lachlan-Cope TA, Carleton AM, Jones PD, Lagun V, Reid PA, Iagovkina S. 2005. Antarctic climate change during the last 50 years. Int J Climatol 25:279–294. doi:10.1002/joc.1130.

[2] Ducklow HW, Baker K, Martinson DG, Quetin LB, Ross RM, Smith RC, Stammerjohn SE, Vernet M, Fraser W. 2007. Marine pelagic ecosystems: the West Antarctic Peninsula. Philos Trans R Soc Lond B Biol Sci 362:67–94. doi:10.1098/rstb.2006.1955. 17405208 PMC1764834

[3] Thiele D, Chester ET, Moore SE, Širovic A, Hildebrand JA, Friedlaender AS. 2004. Seasonal variability in whale encounters in the Western Antarctic Peninsula. Deep Res Part II Top Stud Oceanogr 51:2311–2325. doi:10.1016/j.dsr2.2004.07.007.

[4] Ware C, Friedlaender AS, Nowacek DP. 2011. Shallow and deep lunge feeding of humpback whales in fjords of the West Antarctic Peninsula. Mar Mammal Sci 27:587–605. doi:10.1111/j.1748-7692.2010.00427.x.

[5] Bowman JS, Van Mooy BAS, Lowenstein DP, Fredricks HF, Hansel CM, Gast R, et al. 2021. Whole community metatranscriptomes and lipidomes reveal diverse responses among Antarctic phytoplankton to changing ice conditions. Front Mar Sci 8:593566. doi:10.3389/fmars.2021.593566.

[6] Lin Y, Cassar N, Marchetti A, Moreno C, Ducklow H, Li Z. 2017. Specific eukaryotic plankton are good predictors of net community production in the Western Antarctic Peninsula. Sci Rep 7:14845. doi:10.1038/s41598-017-14109-1. 29093494 PMC5665988

[7] Garibotti IA, Vernet M, Kozlowski WA, Ferrario ME. 2003. Composition and biomass of phytoplankton assemblages in coastal Antarctic waters: a comparison of chemotaxonomic and microscopic analyses. Mar Ecol Prog Ser 247:27–42. doi:10.3354/meps247027.

[8] Garibotti IA, Vernet M, Ferrario ME. 2005. Annually recurrent phytoplanktonic assemblages during summer in the seasonal ice zone west of the Antarctic Peninsula (Southern Ocean). Deep Res Part I Oceanogr Res Pap 52:1823–1841. doi:10.1016/j.dsr.2005.05.003.

[9] Mendes CRB, Tavano VM, Leal MC, de Souza MS, Brotas V, Garcia CAE. 2013. Shifts in the dominance between diatoms and cryptophytes during three late summers in the Bransfield Strait (Antarctic Peninsula). Polar Biol 36:537–547. doi:10.1007/s00300-012-1282-4.

[10] Wright SW, Ishikawa A, Marchant HJ, Davidson AT, Van Den Enden RL, Nash GV. 2009. Composition and significance of picophytoplankton in Antarctic waters. Polar Biol 32:797–808. doi:10.1007/s00300-009-0582-9.

[11] Rodriguez F, Varela M, Zapata M. 2002. Phytoplankton assemblages in the Gerlache and Bransfield Straits (Antarctic Peninsula) determined by light microscopy and CHEMTAX analysis of HPLC pigment data. Deep Res Part II Top Stud Oceanogr 49:723–747. doi:10.1016/S0967-0645(01)00121-7.

[12] Mascioni M, Almandoz GO, Cefarelli AO, Cusick A, Ferrario ME, Vernet M. 2019. Phytoplankton composition and bloom formation in unexplored nearshore waters of the western Antarctic Peninsula. Polar Biol 42:1859–1872. doi:10.1007/s00300-019-02564-7.

[13] Mendes CRB, Tavano VM, Dotto TS, Kerr R, de Souza MS, Garcia CAE, Secchi ER. 2018. New insights on the dominance of cryptophytes in Antarctic coastal waters: a case study in Gerlache Strait. Deep Sea Res Part II Top Stud Oceanogr 149:161–170. doi:10.1016/j.dsr2.2017.02.010.

[14] Luria CM, Ducklow HW, Amaral-Zettler LA. 2014. Marine bacterial, archaeal and eukaryotic diversity and community structure on the continental shelf of the western Antarctic Peninsula. Aquat Microb Ecol 73:107–121. doi:10.3354/ame01703.

[15] Abele D, Vazquez S, Buma AGJ, Hernandez E, Quiroga C, Held C, Frickenhaus S, Harms L, Lopez JL, Helmke E, Mac Cormack WP. 2017. Pelagic and benthic communities of the Antarctic ecosystem of Potter Cove: genomics and ecological implications. Mar Genomics 33:1–11. doi:10.1016/j.margen.2017.05.001. 28479280

[16] Rozema PD, Biggs T, Sprong PAA, Buma AGJ, Venables HJ, Evans C, Meredith MP, Bolhuis H. 2017. Summer microbial community composition governed by upper-ocean stratification and nutrient availability in northern Marguerite Bay, Antarctica. Deep Res Part II Top Stud Oceanogr 139:151–166. doi:10.1016/j.dsr2.2016.11.016.

[17] Fuentes S, Arroyo JI, Rodríguez-Marconi S, Masotti I, Alarcón-Schumacher T, Polz MF, Trefault N, De la Iglesia R, Díez B. 2019. Summer phyto- and bacterioplankton communities during low and high productivity scenarios in the Western Antarctic Peninsula. Polar Biol 42:159–169. doi:10.1007/s00300-018-2411-5.

[18] Trefaut N, De la Iglesia R, Moreno-Pino M, Lopes dos Santos A, Gerikas Ribeiro C, Cristi A, Marie D, Vaulot D. 2021. Annual phytoplankton dynamics in coastal waters from Fildes Bay, Western Antarctic Peninsula. Sci Rep 11:1368. doi:10.1038/s41598-020-80568-8. 33446791 PMC7809266

[19] Brown MS, Bowman JS, Lin Y, Feehan CJ, Moreno CM, Cassar N, Marchetti A, Schofield OM. 2021. Low diversity of a key phytoplankton group along the West Antarctic Peninsula. Limnol Oceanogr 66:2470–2480. doi:10.1002/lno.11765.

[20] Howe JA, Austin WEN, Forwick M, Paetzel M, Harland REX, Cage AG. 2010. Fjord systems and archives: a review. Geol Soc London, Spec Publ 344:5–15. doi:10.1144/SP344.2.

[21] Pan B, Vernet V, Manck L, Forsch K, Ekern L, Mascioni M, Barbeau KA, Almandoz GO, Orona AJ. 2020. Environmental drivers on phytoplankton taxonomic composition in an Antarctic fjord. Prog Oceanogr 183:102295. doi:10.1016/j.pocean.2020.102295.

[22] Grange LJ, Smith CR. 2013. Megafaunal communities in rapidly warming fjords along the West Antarctic Peninsula: hotspots of abundance and beta diversity. PLoS One 8:e77917. doi:10.1371/journal.pone.0077917. 24312442 PMC3848936

[23] Mascioni M, Almandoz GO, Ekern L, Pan BJ, Vernet M. 2021. Microplanktonic diatom assemblages dominated the primary production but not the biomass in an Antarctic fjord. J Mar Syst 224:103624. doi:10.1016/j.jmarsys.2021.103624.

[24] Amaral-Zettler LA, McCliment EA, Ducklow HW, Huse SM. 2009. A method for studying protistan diversity using massively parallel sequencing of V9 hypervariable regions of small-subunit ribosomal RNA Genes. PLoS One 4. doi:10.1371/journal.pone.0006372. PMC2711349 19633714

[25] Needham DM, Fuhrman JA. 2016. Pronounced daily succession of phytoplankton, archaea and bacteria following a spring bloom. Nat Microbiol 1:16005. doi:10.1038/nmicrobiol.2016.5. 27572439

[26] Choi CJ, Jimenez V, Needham DM, Poirier C, Bachy C, Alexander H, Wilken S, Chavez FP, Sudek S, Giovannoni SJ, Worden AZ. 2020. Seasonal and geographical transitions in eukaryotic phytoplankton community structure in the Atlantic and Pacific Oceans. Front Microbiol 11:542372. doi:10.3389/fmicb.2020.542372. 33101224 PMC7554337

[27] Taylor DL, Lee CC. 1971. A new cryptomonad from antarctica: *Cryptomonas cryophila* sp. nov. Archiv Mikrobiol 75:269–280. doi:10.1007/BF00407688.

[28] Zhou M, Niiler PP, Hu JH. 2002. Surface currents in the Bransfield and Gerlache Straits, Antarctica. Deep Res Part I Oceanogr Res Pap 49:267–280. doi:10.1016/S0967-0637(01)00062-0.

[29] Wideman JG, Monier A, Rodríguez-Martínez R, Leonard G, Cook E, Poirier C, Maguire F, Milner DS, Irwin NAT, Moore K, Santoro AE, Keeling PJ, Worden AZ, Richards TA. 2020. Unexpected mitochondrial genome diversity revealed by targeted single-cell genomics of heterotrophic flagellated protists. Nat Microbiol 5:154–165. doi:10.1038/s41564-019-0605-4. 31768028

[30] Keeling PJ, Burki F. 2019. Progress towards the tree of eukaryotes. Curr Biol 29:R808–R817. doi:10.1016/j.cub.2019.07.031. 31430481

[31] Olson RJ, Zettler ER, Anderson OK. 1989. Discrimination of eukaryotic phytoplankton cell types from light scatter and autofluorescence properties measured by flow cytometry. Cytometry 10:636–643. doi:10.1002/cyto.990100520. 2776580

[32] Vergin KL, Beszteri B, Monier A, Thrash JC, Temperton B, Treusch AH, Kilpert F, Worden AZ, Giovannoni SJ. 2013. High-resolution SAR11 ecotype dynamics at the Bermuda Atlantic time-series study site by phylogenetic placement of pyrosequences. ISME J 7:1322–1332. doi:10.1038/ismej.2013.32. 23466704 PMC3695298

[33] Smith WO, Lancelot C. 2004. Bottom-up versus top-down control in phytoplankton of the Southern Ocean. Antartic Science 16:531–539. doi:10.1017/S0954102004002305.

[34] Sudek S, Everroad RC, Gehman A-LM, Smith JM, Poirier CL, Chavez FP, Worden AZ. 2015. Cyanobacterial distributions along a physico-chemical gradient in the Northeastern Pacific Ocean. Environ Microbiol 17:3692–3707. doi:10.1111/1462-2920.12742. 25522910

[35] Bolaños LM, Karp-Boss L, Choi CJ, Worden AZ, Graff JR, Haëntjens N, Chase AP, Della Penna A, Gaube P, Morison F, Menden-Deuer S, Westberry TK, O’Malley RT, Boss E, Behrenfeld MJ, Giovannoni SJ. 2020. Small phytoplankton dominate western North Atlantic biomass. ISME J 14:1663–1674. doi:10.1038/s41396-020-0636-0. 32231247 PMC7305139

[36] Round FE, Crawford DW, Mann DG. 1990. The diatoms: biology and morphology of the genera. Cambridge University Press, Cambridge, United Kingdom.

[37] Theriot EC, Ashworth M, Ruck E, Nakov T, Jansen RK. 2010. A preliminary multigene phylogeny of the diatoms (Bacillariophyta): challenges for future research. Plecevo 143:278–296. doi:10.5091/plecevo.2010.418.

[38] Ichinomiya M, Dos Santos AL, Gourvil P, Yoshikawa S, Kamiya M, Ohki K, Audic S, de Vargas C, Noël M-H, Vaulot D, Kuwata A. 2016. Diversity and oceanic distribution of Parmales and Bolidophytes, a group closely related to diatoms. ISME J 10:2419–2434. doi:10.1038/ismej.2016.38. 27003244 PMC5030691

[39] Tajima N, Saitoh K, Sato S, Maruyama F, Ichinomiya M, Yoshikawa S, Kurokawa K, Ohta H, Tabata S, Kuwata A, Sato N. 2016. Sequencing and analysis of the complete organellar genomes of Parmales, a closely related group to Bacillariophyta (diatoms). Curr Genet 62:887–896. doi:10.1007/s00294-016-0598-y. 27091756

[40] van den Hoff J, Bell E, Whittock L. 2020. Dimorphism in the Antarctic cryptophyte *Geminigera cryophila* (Cryptophyceae). J Phycol 56:1028–1038. doi:10.1111/jpy.13004. 32289881

[41] Hoef-Emden K. 2008. Molecular phylogeny of phycocyanin-containing cryptophytes: evolution of biliproteins and geographical distribution. J Phycol 44:985–993. doi:10.1111/j.1529-8817.2008.00530.x. 27041617

[42] Laza-Martínez A, Arluzea J, Miguel I, Orive E. 2012. Morphological and molecular characterization of *Teleaulax gracilis* sp. nov. and *T. minuta* sp. nov. (Cryptophyceae). Phycologia 51:649–661. doi:10.2216/11-044.1.

[43] Ibarbalz FM, Henry N, Brandão MC, Martini S, Busseni G, Byrne H, Coelho LP, Endo H, Gasol JM, Gregory AC, Mahé F, Rigonato J, Royo-Llonch M, Salazar G, Sanz-Sáez I, Scalco E, Soviadan D, Zayed AA, Zingone A, Labadie K, Ferland J, Marec C, Kandels S, Picheral M, Dimier C, Poulain J, Pisarev S, Carmichael M, Pesant S, Babin M, Boss E, Iudicone D, Jaillon O, Acinas SG, Ogata H, Pelletier E, Stemmann L, Sullivan MB, Sunagawa S, Bopp L, de Vargas C, Karp-Boss L, Wincker P, Lombard F, Bowler C, Zinger L, *Tara* Oceans Coordinators. 2019. Global trends in marine plankton diversity across kingdoms of life. Cell 179:1084–1097.e21. doi:10.1016/j.cell.2019.10.008. 31730851 PMC6912166

[44] Šlapeta J, Moreira D, López-García P. 2005. The extent of protist diversity: insights from molecular ecology of freshwater eukaryotes. Proc Biol Sci 272:2073–2081. doi:10.1098/rspb.2005.3195. 16191619 PMC1559898

[45] Arroyo AS, López-Escardó D, Kim E, Ruiz-Trillo I, Najle SR. 2018. Novel diversity of deeply branching holomycota and unicellular holozoans revealed by metabarcoding in Middle Paraná River, Argentina. Front Ecol Evol 6:99. doi:10.3389/fevo.2018.00099.

[46] Lovejoy C, Massana R, Pedro C. 2006. Diversity and distribution of marine microbial eukaryotes in the Arctic Ocean and adjacent seas diversity and distribution of marine microbial eukaryotes in the Arctic Ocean and adjacent seas. Appl Environ Microbiol 72:3085–3095. doi:10.1128/AEM.72.5.3085-3095.2006. 16672445 PMC1472370

[47] Bachy C, López-García P, Vereshchaka A, Moreira D. 2011. Diversity and vertical distribution of microbial eukaryotes in the snow, sea ice and seawater near the North Pole at the end of the polar night. Front Microbiol 2:106. 21833337 10.3389/fmicb.2011.00106 PMC3153057

[48] Wolf C, Kilias E, Metfies K. 2015. Protists in the polar regions: comparing occurrence in the Arctic and Southern oceans using pyrosequencing. Polar Res 34. doi:10.3402/polar.v34.23225.

[49] Luo W, Li H, Gao S, Yu Y, Lin L, Zeng Y. 2016. Molecular diversity of microbial eukaryotes in sea water from Fildes Peninsula, King George Island, Antarctica. Polar Biol 39:605–616. doi:10.1007/s00300-015-1815-8.

[50] Zoccarato L, Pallavicini A, Cerino F, Fonda Umani S, Celussi M. 2016. Water mass dynamics shape Ross Sea protist communities in mesopelagic and bathypelagic layers. Prog Oceanogr 149:16–26. doi:10.1016/j.pocean.2016.10.003.

[51] Zhang F, Cao S, Gao Y, He J. 2019. Distribution and environmental correlations of picoeukaryotes in an arctic fjord (Kongsfjorden, Svalbard) during the summer. Polar Res 38.

[52] Piwosz K, Walkusz W, Hapter R, Wieczorek P, Hop H, Wiktor J. 2009. Comparison of productivity and phytoplankton in a warm (Kongsfjorden) and a cold (Hornsund) Spitsbergen fjord in mid-summer 2002. Polar Biol 32:549–559. doi:10.1007/s00300-008-0549-2.

[53] Wlodarska-Kowalczuk M, Pearson TH, Kendall MA. 2005. Benthic response to chronic natural physical disturbance by glacial sedimentation in an Arctic fjord. Mar Ecol Prog Ser 303:31–41. doi:10.3354/meps303031.

[54] Keigwin LD, Jones GA, Lehman SJ, Boyle EA. 1991. Deglacial meltwater discharge, North Atlantic deep circulation, and abrupt climate change. J Geophys Res 96.

[55] Kubiszyn AM, Piwosz K, Wiktor JM, Wiktor JM. 2014. The effect of inter-annual Atlantic water inflow variability on the planktonic protist community structure in the West Spitsbergen waters during the summer. J Plankton Res 36:1190–1203. doi:10.1093/plankt/fbu044.

[56] Lin YC, Campbell T, Chung CC, Gong GC, Chiang KP, Worden AZ. 2012. Distribution patterns and phylogeny of marine stramenopiles in the North Pacific Ocean. Appl Environ Microbiol 78:3387–3399. doi:10.1128/AEM.06952-11. 22344659 PMC3346489

[57] Massana R, Del Campo J, Sieracki ME, Audic S, Logares R. 2014. Exploring the uncultured microeukaryote majority in the oceans: reevaluation of ribogroups within stramenopiles. ISME J 8:854–866. doi:10.1038/ismej.2013.204. 24196325 PMC3960543

[58] López-García P, Rodríguez-Valera F, Pedrós-Alió C, Moreira D. 2001. Unexpected diversity of small eukaryotes in deep-sea Antarctic plankton. Nature 409:603–606. doi:10.1038/35054537. 11214316

[59] Gast RJ, Moran DM, Beaudoin DJ, Blythe JN, Dennett MR, Caron DA. 2006. Abundance of a novel dinoflagellate phylotype in the Ross Sea, Antarctica. J Phycol 42:233–242. doi:10.1111/j.1529-8817.2006.00183.x.

[60] Torstensson A, Dinasquet J, Chierici M, Fransson A, Riemann L, Wulff A. 2015. Physicochemical control of bacterial and protist community composition and diversity in Antarctic sea ice. Environ Microbiol 17:3869–3881. doi:10.1111/1462-2920.12865. 25845501

[61] Monier A, Worden AZ, Richards TA. 2016. Phylogenetic diversity and biogeography of the Mamiellophyceae lineage of eukaryotic phytoplankton across the oceans. Environ Microbiol Rep 8:461–469. doi:10.1111/1758-2229.12390. 26929141

[62] Selz V, Lowry KE, Lewis KM, Joy-Warren HL, Poll W, Nirmel S, Tong A, Arrigo KR. 2018. Distribution of *Phaeocystis antarctica*-dominated sea ice algal communities and their potential to seed phytoplankton across the western Antarctic Peninsula in spring. Mar Ecol Prog Ser 586:91–112. doi:10.3354/meps12367.

[63] Guillou L, Chrétiennot-Dinet M-J, Medlin LK, Claustre H, Goër S. L-d, Vaulot D. 1999. Bolidomonas: a new genus with two species belonging to a new algal class, the Bolidophyceae (Heterokonta). J Phycol 35:368–381. doi:10.1046/j.1529-8817.1999.3520368.x.

[64] Gast RJ, Moran DM, Dennett MR, Caron DA. 2007. Kleptoplasty in an Antarctic dinoflagellate: caught in evolutionary transition? Environ Microbiol 9:39–45. doi:10.1111/j.1462-2920.2006.01109.x. 17227410

[65] Worden AZ, Follows MJ, Giovannoni SJ, Wilken S, Zimmerman AE, Keeling PJ. 2015. Rethinking the marine carbon cycle: factoring in the multifarious lifestyles of microbes. Science (80-) 347 doi:10.1126/science.1257594. 25678667

[66] Bell EM, Laybourn-Parry J. 2003. Mixotrophy in the Antarctic phytoflagellate, *Pyramimonas gelidicola* (Chlorophyta: Prasinophyceae). J Phycol 39:644–649. doi:10.1046/j.1529-8817.2003.02152.x.

[67] Hoff J, Burton HR, Vesk M. 1989. An encystment stage, bearing a new scale type of the antartic prasinophyte *Pyramimonas gelidicola* and its paleolimnological and taxonomic significance. J Phycol 25:446–454. doi:10.1111/j.1529-8817.1989.tb00249.x.

[68] Delmont TO, Murat Eren A, Vineis JH, Post AF. 2015. Genome reconstructions indicate the partitioning of ecological functions inside a phytoplankton bloom in the Amundsen Sea. Front Microbiol 6:1090. 26579075 10.3389/fmicb.2015.01090 PMC4620155

[69] Simmons MP, Bachy C, Sudek S, van Baren MJ, Sudek L, Ares M, Worden AZ. 2015. Intron invasions trace algal speciation and reveal nearly identical Arctic and Antarctic *Micromonas* populations. Mol Biol Evol 32:2219–2235. doi:10.1093/molbev/msv122. 25998521 PMC4540971

[70] Lundesgaard Ø, Winsor P, Truffer M, Merrifield M, Powell B, Statscewich H, Eidam E, Smith CR. 2020. Hydrography and energetics of a cold subpolar fjord: Andvord Bay, western Antarctic Peninsula. Prog Oceanogr 181:102224. doi:10.1016/j.pocean.2019.102224.

[71] Malviya S, Scalco E, Audic S, Vincent F, Veluchamy A, Poulain J, Wincker P, Iudicone D, de Vargas C, Bittner L, Zingone A, Bowler C. 2016. Insights into global diatom distribution and diversity in the world’s ocean. Proc Natl Acad Sci USA 113:E1516–25. doi:10.1073/pnas.1509523113. 26929361 PMC4801293

[72] Ekern L. 2017. Assessing seasonal primary production in Andvord Bay, Antarctica. University of California, San Diego, CA.

[73] Altenburger A, Blossom HE, Garcia-Cuetos L, Jakobsen HH, Carstensen J, Lundholm N. 2020. Dimorphism in cryptophytes—the case of *Teleaulax amphioxeia*/*Plagioselmis prolonga* and its ecological implications. Sci Adv 6:eabb1611. doi:10.1126/sciadv.abb1611. 32917704 PMC7486100

[74] Biggs TEG, Alvarez-Fernandez S, Evans C, Mojica KDA, Rozema PD, Venables HJ, Pond DW, Brussaard CPD. 2019. Antarctic phytoplankton community composition and size structure: importance of ice type and temperature as regulatory factors. Polar Biol 42:1997–2015. doi:10.1007/s00300-019-02576-3.

[75] Schofield O, Saba G, Coleman K, Carvalho F, Couto N, Ducklow H, Finkel Z, Irwin A, Kahl A, Miles T, Montes-Hugo M, Stammerjohn S, Waite N. 2017. Decadal variability in coastal phytoplankton community composition in a changing West Antarctic Peninsula. Deep Res Part I Oceanogr Res Pap 124:42–54. doi:10.1016/j.dsr.2017.04.014.

[76] Calleja ML, Kerhervé P, Bourgeois S, Kędra M, Leynaert A, Devred E, Babin M, Morata N. 2017. Effects of increase glacier discharge on phytoplankton bloom dynamics and pelagic geochemistry in a high Arctic fjord. Prog Oceanogr 159:195–210. doi:10.1016/j.pocean.2017.07.005.

[77] Piquet AMT, Van De Poll WH, Visser RJW, Wiencke C, Bolhuis H, Buma AGJ. 2014. Springtime phytoplankton dynamics in Arctic Krossfjorden and Kongsfjorden (Spitsbergen) as a function of glacier proximity. Biogeosciences 11:2263–2279. doi:10.5194/bg-11-2263-2014.

[78] Darling KF, Wade CM, Stewart IA, Kroon D, Dingle R, Brown AJ. 2000. Molecular evidence for genetic mixing of Arctic and Antarctic subpolar populations of planktonic foraminifers. Nature 405:43–47. doi:10.1038/35011002. 10811211

[79] Cuvelier ML, Allen AE, Monier A, McCrow JP, Messié M, Tringe SG, Woyke T, Welsh RM, Ishoey T, Lee J-H, Binder BJ, DuPont CL, Latasa M, Guigand C, Buck KR, Hilton J, Thiagarajan M, Caler E, Read B, Lasken RS, Chavez FP, Worden AZ. 2010. Targeted metagenomics and ecology of globally important uncultured eukaryotic phytoplankton. Proc Natl Acad Sci USA 107:14679–14684. doi:10.1073/pnas.1001665107. 20668244 PMC2930470

[80] Demir-Hilton E, Sudek S, Cuvelier ML, Gentemann CL, Zehr JP, Worden AZ. 2011. Global distribution patterns of distinct clades of the photosynthetic picoeukaryote *Ostreococcus*. ISME J 5:1095–1107. doi:10.1038/ismej.2010.209. 21289652 PMC3146286

[81] Daims H, Brühl A, Amann R, Schleifer KH, Wagner M. 1999. The domain-specific probe EUB338 is insufficient for the detection of all bacteria: development and evaluation of a more comprehensive probe set. Syst Appl Microbiol 22:434–444. doi:10.1016/S0723-2020(99)80053-8. 10553296

[82] Bolyen E, Rideout JR, Dillon MR, Bokulich NA, Abnet CC, Al-Ghalith GA, Alexander H, Alm EJ, Arumugam M, Asnicar F, Bai Y, Bisanz JE, Bittinger K, Brejnrod A, Brislawn CJ, Brown CT, Callahan BJ, Caraballo-Rodríguez AM, Chase J, Cope EK, Da Silva R, Diener C, Dorrestein PC, Douglas GM, Durall DM, Duvallet C, Edwardson CF, Ernst M, Estaki M, Fouquier J, Gauglitz JM, Gibbons SM, Gibson DL, Gonzalez A, Gorlick K, Guo J, Hillmann B, Holmes S, Holste H, Huttenhower C, Huttley GA, Janssen S, Jarmusch AK, Jiang L, Kaehler BD, Kang KB, Keefe CR, Keim P, Kelley ST, Knights D, et al. 2019. Reproducible, interactive, scalable and extensible microbiome data science using QIIME 2. Nat Biotechnol 37:852–857. doi:10.1038/s41587-019-0209-9. 31341288 PMC7015180

[83] Guillou L, Bachar D, Audic S, Bass D, Berney C, Bittner L. 2013. The Protist Ribosomal Reference database (PR^2^): a catalog of unicellular eukaryote small sub-unit rRNA sequences with curated taxonomy. Nucleic Acids Res 41:597–604. 10.1093/nar/gks1160 PMC3531120 23193267

[84] Stamatakis A. 2006. RAxML-VI-HPC: maximum likelihood-based phylogenetic analyses with thousands of taxa and mixed models. Bioinformatics 22:2688–2690. doi:10.1093/bioinformatics/btl446. 16928733

[85] Katoh K, Standley DM. 2013. MAFFT multiple sequence alignment software version 7: improvements in performance and usability. Mol Biol Evol 30:772–780. doi:10.1093/molbev/mst010. 23329690 PMC3603318

[86] Barbera P, Kozlov AM, Czech L, Morel B, Darriba D, Flouri T, Stamatakis A. 2019. EPA-ng: massively parallel evolutionary placement of genetic sequences. Syst Biol 68:365–369. doi:10.1093/sysbio/syy054. 30165689 PMC6368480

[87] Junier T, Zdobnov EM. 2010. The Newick utilities: high-throughput phylogenetic tree processing in the UNIX shell. Bioinformatics 26:1669–1670. doi:10.1093/bioinformatics/btq243. 20472542 PMC2887050

[88] Yu G, Smith DK, Zhu H, Guan Y, Lam TTY. 2017. Ggtree: an R package for visualization and annotation of phylogenetic trees with their covariates and other associated data. Methods Ecol Evol 8:28–36. doi:10.1111/2041-210X.12628.

[89] Sun J, Liu D. 2003. Geometric models for calculating cell biovolume and surface area for phytoplankton. J Plankton Res 25:1331–1346. doi:10.1093/plankt/fbg096.

[90] Menden-Deuer S, Lessard EJ. 2000. Carbon to volume relationships for dinoflagellates, diatoms, and other protist plankton. Limnol Oceanogr 45:569–579. doi:10.4319/lo.2000.45.3.0569.

[91] Worden AZ, Nolan JK, Palenik B. 2004. Assessing the dynamic and ecology of marine picoplankton: the importance of eukaryotic component. Limnol Oceanogr 49:168–179. doi:10.4319/lo.2004.49.1.0168.

[92] Moon-Van Der Staay SY, Van Der Staay GWM, Guillou L, Vaulot D, Claustre H, Médlin LK. 2000. Abundance and diversity of prymnesiophytes in the picoplankton community from the equatorial Pacific Ocean inferred from 18S rDNA sequences. Limnol Oceanogr 45:98–109. doi:10.4319/lo.2000.45.1.0098.

[93] Altschul SF, Gish W, Miller W, Myers EW, Lipman DJ. 1990. Basic local alignment search tool. J Mol Biol 215:403–410. doi:10.1016/S0022-2836(05)80360-2. 2231712

[94] Daugbjerg N, Norlin A, Lovejoy C. 2018. *Baffinella frigidus* gen. et sp. nov. (Baffinellaceae fam. nov., Cryptophyceae) from Baffin Bay: morphology, pigment profile, phylogeny, and growth rate response to three abiotic factors. J Phycol 54:665–680. doi:10.1111/jpy.12766. 30043990

[95] Capella-Gutiérrez S, Silla-Martínez JM, Gabaldón T. 2009. trimAl: a tool for automated alignment trimming in large-scale phylogenetic analyses. Bioinformatics 25:1972–1973. doi:10.1093/bioinformatics/btp348. 19505945 PMC2712344

[96] Stamatakis A. 2015. Using RAxML to infer phylogenies. Curr Protoc Bioinformatics 51:6.14.1–6.14.14. 26334924 10.1002/0471250953.bi0614s51

[97] Ronquist F, Teslenko M, van der Mark P, Ayres DL, Darling A, Höhna S, Larget B, Liu L, Suchard MA, Huelsenbeck JP. 2012. MrBayes 3.2: efficient Bayesian phylogenetic inference and model choice across a large model space. Syst Biol 61:539–542. doi:10.1093/sysbio/sys029. 22357727 PMC3329765

