# Supplementary figures and images for: Spatiotemporal Variations in Antarctic Protistan Communities Highlight Phytoplankton Diversity and Seasonal Dominance by a Novel Cryptophyte Lineage
Source: mBio. 2021 Dec 14;12(6):e02973-21. doi: 10.1128/mBio.02973-21 (PMC8669470; doi:10.1128/mBio.02973-21)

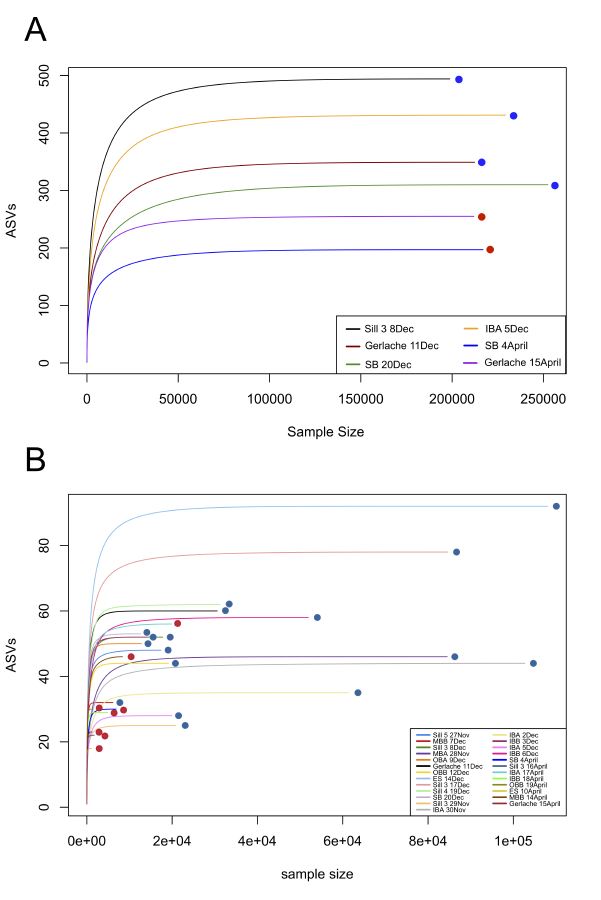

Supplement: FIG S1 [file mbio.02973-21-sf001.tif]

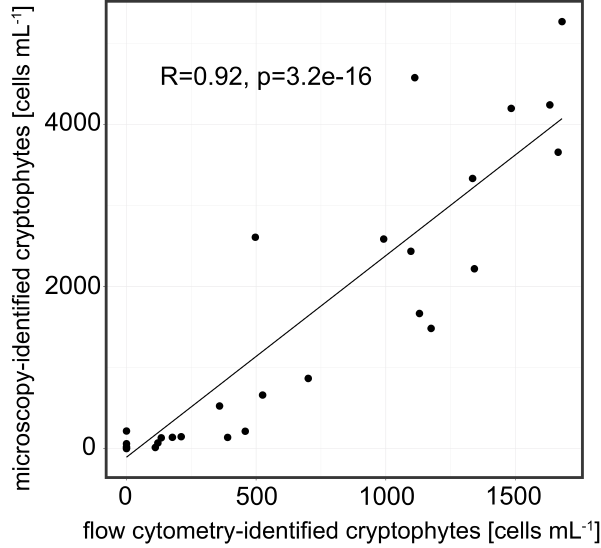

Supplement: FIG S2 [file mbio.02973-21-sf002.tif]

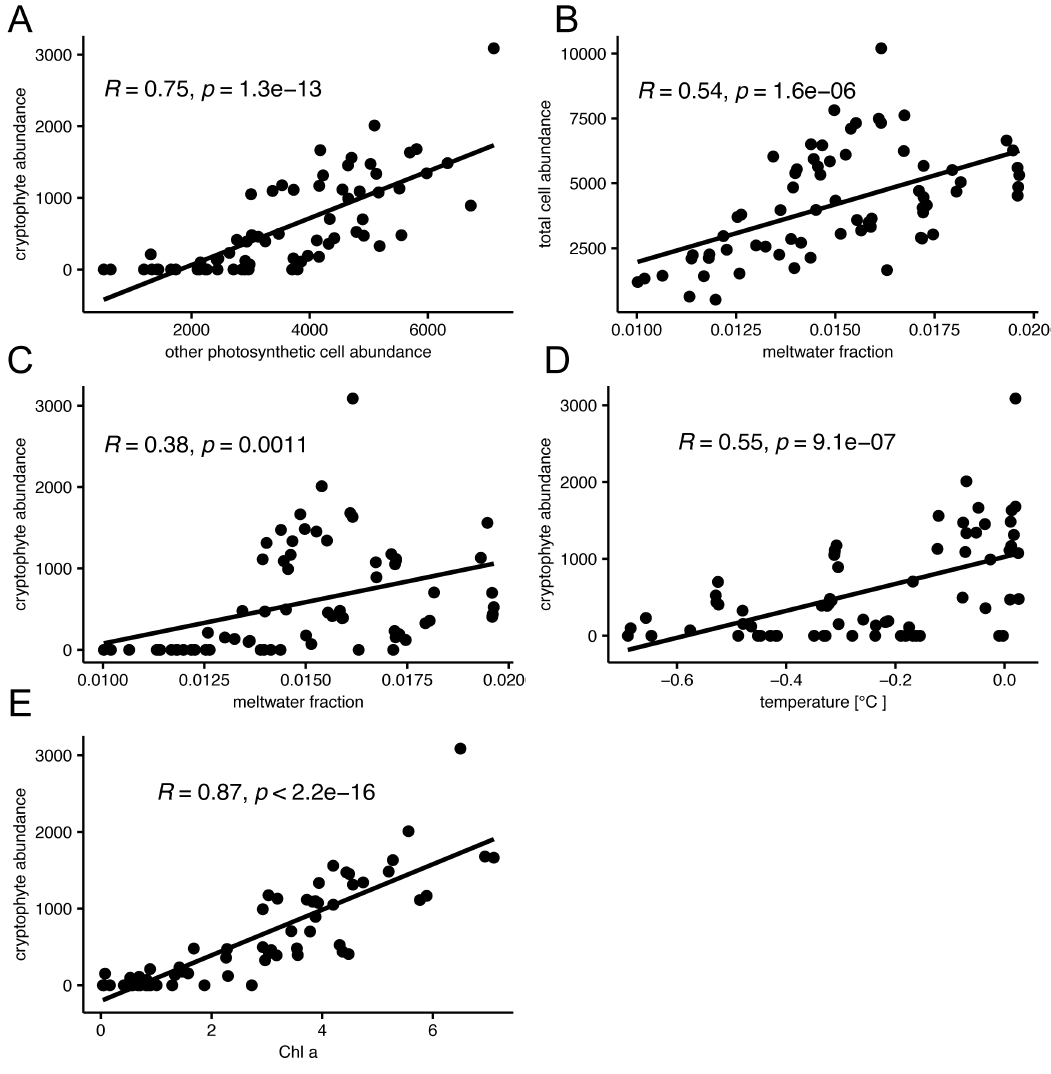

Supplement: FIG S3 [file mbio.02973-21-sf003.tif]

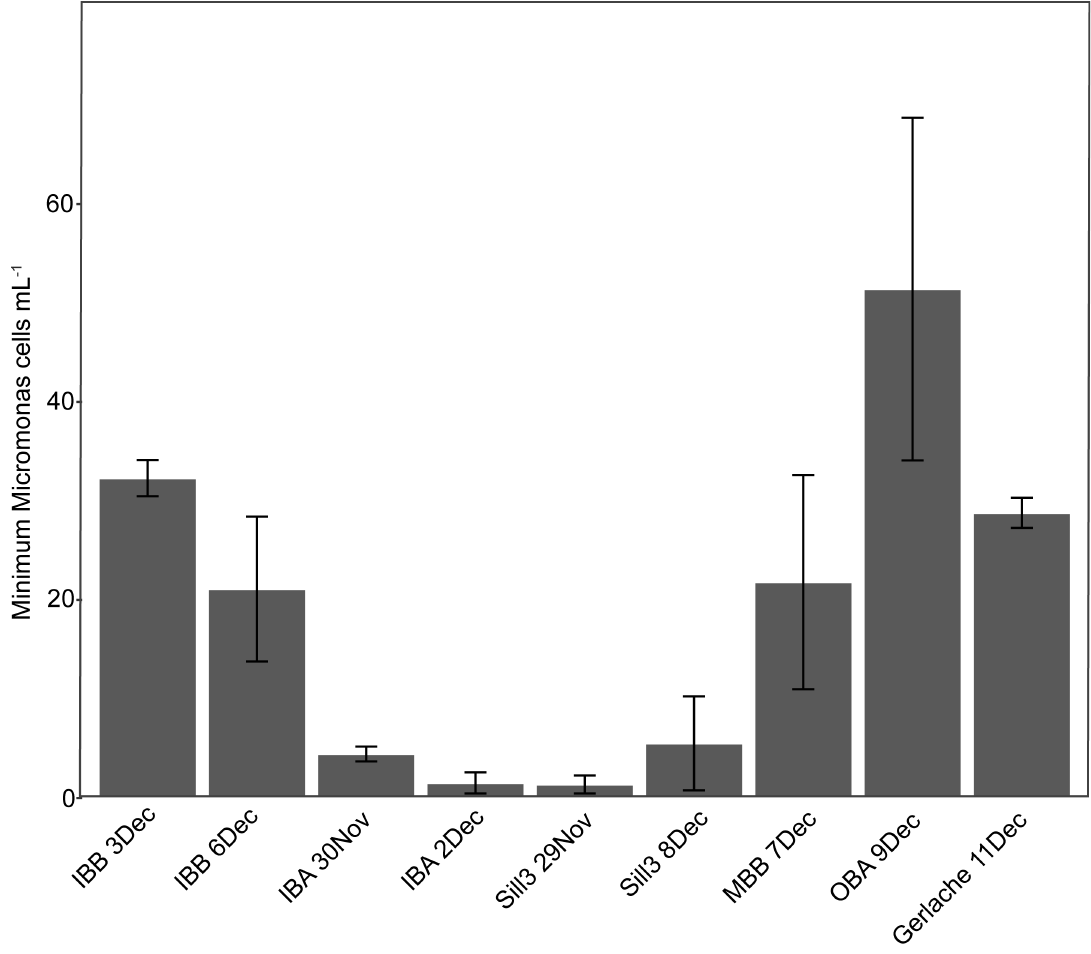

Supplement: FIG S4 [file mbio.02973-21-sf004.tif]

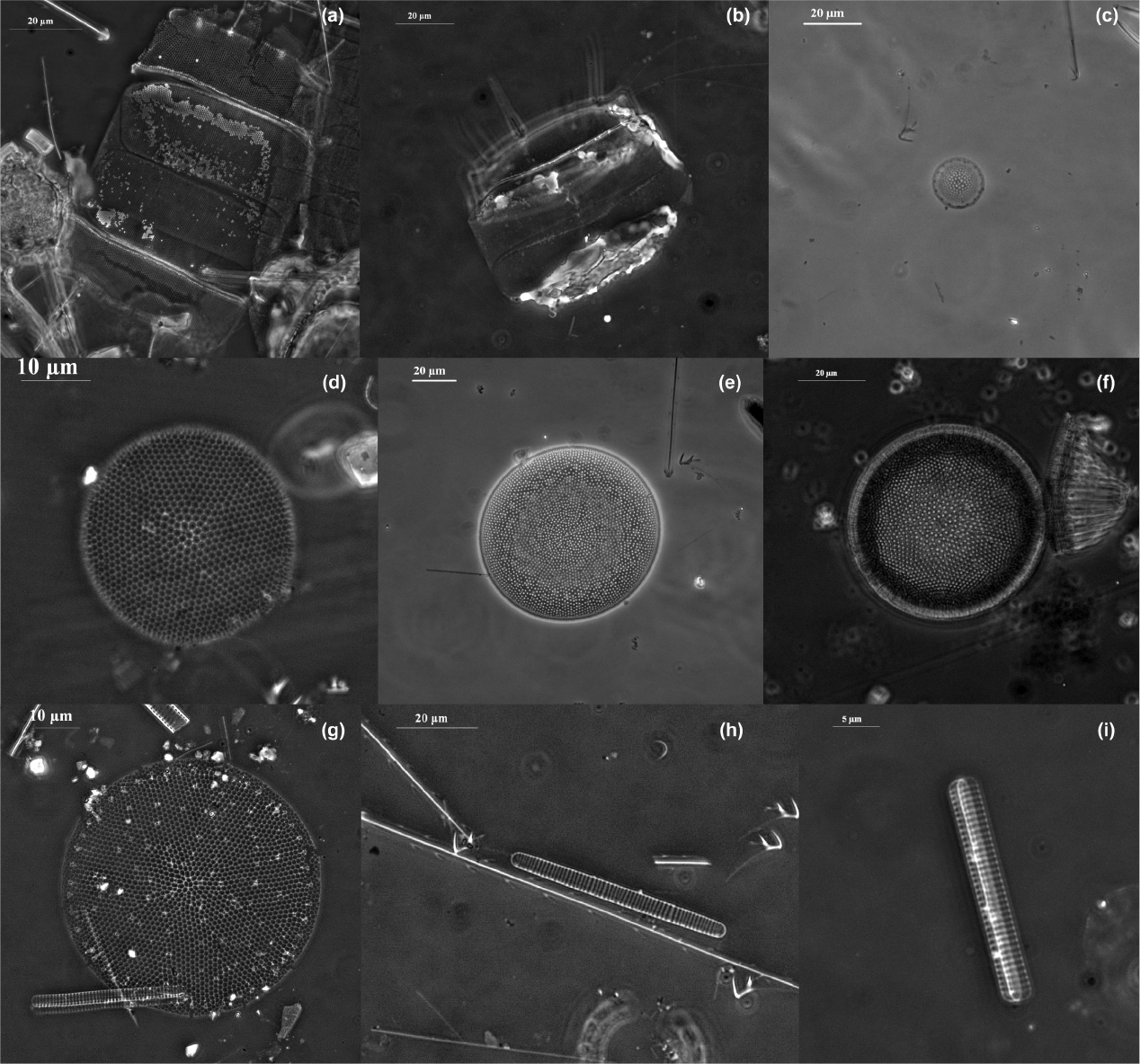

Supplement: FIG S5 [file mbio.02973-21-sf005.tif]

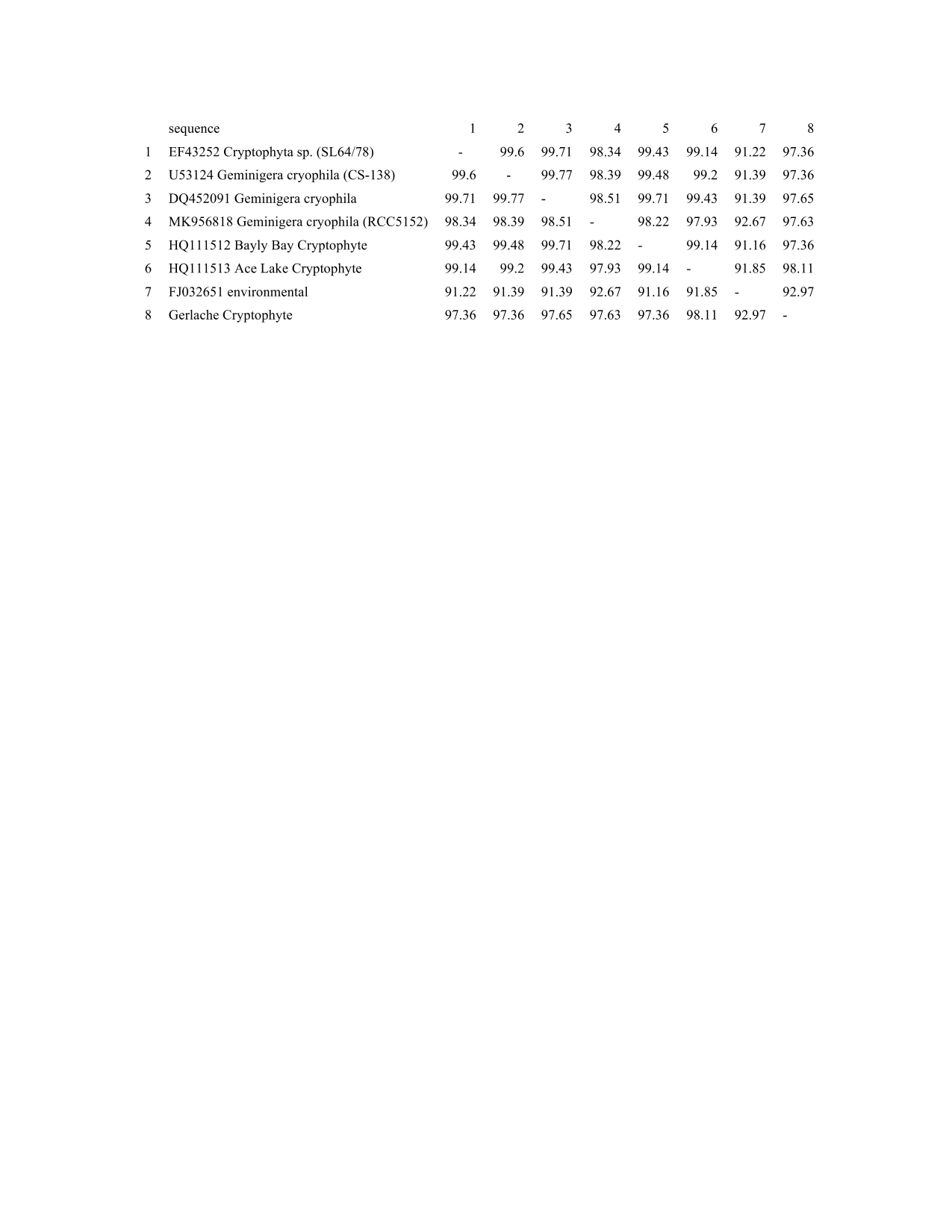

Supplement: FIG S6 [file mbio.02973-21-sf006.tif]

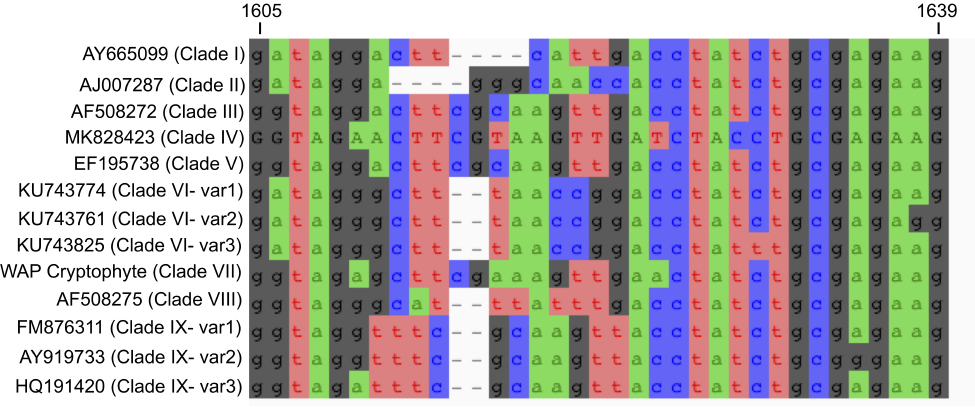

Supplement: FIG S7 [file mbio.02973-21-sf007.tif]
